# Toll-Like Receptor-2 Mediates Diet and/or Pathogen Associated Atherosclerosis: Proteomic Findings

**DOI:** 10.1371/journal.pone.0003204

**Published:** 2008-09-12

**Authors:** Monika Madan, Salomon Amar

**Affiliations:** Department of Periodontology and Oral Biology, School of Dental Medicine, Boston University, Boston, Massachusetts, United States of America; University of Michigan, United States of America

## Abstract

**Background:**

Accumulating evidence implicates a fundamental link between the immune system and atherosclerosis. Toll-like receptors are principal sensors of the innate immune system. Here we report an assessment of the role of the TLR2 pathway in atherosclerosis associated with a high-fat diet and/or bacteria in ApoE^+/−^ mice.

**Methods and Results:**

To explore the role of TLR2 in inflammation- and infection-associated atherosclerosis, 10 week-old ApoE^+/−^-TLR2^+/+^, ApoE^+/−^-TLR2^+/−^ and ApoE^+/−^-TLR2^−/−^ mice were fed either a high fat diet or a regular chow diet. All mice were inoculated intravenously, once per week for 24 consecutive weeks, with 50 µl live *Porphyromonas gingivalis* (*P.g*) (10^7^ CFU) or vehicle (normal saline). Animals were euthanized 24 weeks after the first inoculation. ApoE^+/−^-TLR2^+/+^ mice showed a significant increase in atheromatous lesions in proximal aorta and aortic tree compared to ApoE^+/−^-TLR2^+/−^ and ApoE^+/−^-TLR2^−/−^ mice for all diet conditions. They also displayed profound changes in plaque composition, as evidenced by increased macrophage infiltration and apoptosis, increased lipid content, and decreased smooth muscle cell mass, all reflecting an unstable plaque phenotype. SAA levels from ApoE^+/−^-TLR2^+/+^ mice were significantly higher than from ApoE^+/−^-TLR2^+/−^ and ApoE^+/−^-TLR2^−/−^ mice. Serum cytokine analysis revealed increased levels of pro-inflammatory cytokines in ApoE^+/−^-TLR2^+/+^ mice compared to ApoE^+/−^-TLR2^+/−^ and TLR2^−/−^ mice, irrespective of diet or bacterial challenge. ApoE^+/−^-TLR2^+/+^ mice injected weekly for 24 weeks with FSL-1 (a TLR2 agonist) also demonstrated significant increases in atherosclerotic lesions, SAA and serum cytokine levels compared to ApoE^+/−^-TLR2^−/−^ mice under same treatment condition. Finally, mass-spectrometry (MALDI-TOF-MS) of aortic samples analyzed by 2-dimentional gel electrophoresis differential display, identified 6 proteins upregulated greater than 2-fold in ApoE^+/−^-TLR2^+/+^ mice fed the high fat diet and inoculated with *P.g* compared to any other group.

**Conclusion:**

Genetic deficiency of TLR2 reduces diet- and/or pathogen-associated atherosclerosis in ApoE^+/−^ mice, along with differences in plaque composition suggesting greater structural stability while TLR-2 ligand-specific activation triggers atherosclerosis. The present data offers new insights into the pathophysiological pathways involved in atherosclerosis and paves the way for new pharmacological interventions aimed at reducing atherosclerosis.

## Introduction

Atherosclerosis is a multifactorial chronic inflammatory disease characterized by the accumulation of cells of both the innate and acquired immune systems within the intima of the arterial wall [1], [2]. In atherosclerosis, the normal homeostatic functions of the endothelium are altered, promoting an inflammatory response that results in increased expression of adhesion molecules. This in turn leads to recruitment of leukocytes, including monocytes, which penetrate into the intima, predisposing the vessel wall to lipid accretion [1], [3], [4]. Inflammatory mediators enhance uptake of modified lipoprotein particles and formation of lipid-laden macrophages. The adaptive immune response in atherosclerosis is mediated by T cells that enter the intima and secrete cytokines, which subsequently amplify the inflammatory response and promote the migration and proliferation of intimal smooth muscle cells. [2], [5].

The innate immune system involves several different cell types, most importantly those of the mononuclear phagocyte lineage [6], [7], [8]. Macrophages and endothelial cells (EC) express receptors that recognize a broad range of molecular patterns foreign to the mammalian organism but commonly found on pathogens. These molecules include lipopolysaccharides and lipoproteins from Gram-negative bacteria, peptidoglycan and lipoteichoic acids from Gram-positive bacteria, lipoproteins from mycoplasma, and zymosan from yeast [9]. These pattern-recognition receptors include various scavenger receptors (ScRs) and Toll-like receptors (TLRs).

TLRs are members of a large superfamily containing the interleukin-1 receptors (IL-1R) that share significant homology in their cytoplasmic domain, which is known as the Toll/IL-1R (TIR) domain [10]. Ligation of most TLRs transmits transmembrane signals that activate the NF-κB and mitogen-activated protein kinase (MAPK) pathways [8], [11], [12], [13].

Both *in vitro* and *in vivo* knockout mouse studies have implicated TLRs in neointima formation and intimal hyperplasia involving modulation of inflammatory responses to exogenous and endogenous stimuli [14]. Although TLRs mediate protection against infection, various studies have demonstrated increased expression of TLR1, 2, and 4 in human atherosclerotic lesion, mechanistically linking TLRs, inflammation and atherosclerosis [6], [15], [16] with downstream signaling of TLR directly regulating inflammatory genes. *In vitro* stimulation of TLRs in human fibroblasts with a synthetic fibroblast stimulating lipopeptide (FSL-1; Pam2CGDPKHPKSF) leads to activation of NF-κB and the production of inflammatory cytokines in a MyD88-dependent manner [17], [18].

Two important observations suggest TLR2 as a novel target to consider for therapeutic intervention in atherosclerosis. One is that TLR2 mediates responses to lipoproteins derived from multiple pathogens. Its unique ability to heterodimerize with TLR1 or TLR6 thus results in a relatively broad range of ligand specificity [19], [20], [21] which may contribute to atherogenesis in the context of exposure to a variety of pathogens.

The potential importance of infectious agents of oral/periodontal origin, such as *Porphyromonas gingivalis* (*P.g)* in the development of atherosclerosis has recently been described [22], [23], [24], [25], [26], [27]. To our knowledge, the direct role of TLR2 in the bacteria-enhanced atherogenic process had never been addressed and warranted further investigation. Therefore, in the work reported here, we examined the effect of genetic deletion of TLR2 on the progression of atherosclerosis driven by a high fat diet and/or *P. g* infection in the ApoE^+/−^ murine model. Stimulation by the specific agonist FSL-1 was used to further establish the role of TLR2 in modulating the progression of diet and/or bacteria enhanced atherosclerosis in mice with normal expression of TLR2.

## Results

### Levels of Glucose, Total Serum Cholesterol, LDL, and HDL

Mice were monitored for metabolic status by measuring blood glucose and lipids. No significant differences in body weight were observed in mice in connection to genotype or treatment, (*e.g*. *P. g*, FSL-1). Total serum cholesterol, LDL, HDL or glucose levels also revealed no significant differences among ApoE^+/−^-TLR2^+/+^, ApoE^+/−^-TLR2^+/−^ and ApoE^+/−^-TLR2^−/−^ mice that received similar treatments and were maintained on a similar diet. However, we observed a tendency of increased cholesterol, LDL and decreased HDL level in ApoE^+/−^-TLR2^+/+^ mice injected with *P. g* as compared to vehicle injected group. Furthermore, the lipid and glucose profiles did not reveal any differences between mice of the same genotype that were injected with *P. g* or FSL-1 (supplemental data: Table S1 and Table S2).

### *En face* and Histomorphometric Analysis of Atheroma Lesions

**Figure 1:**
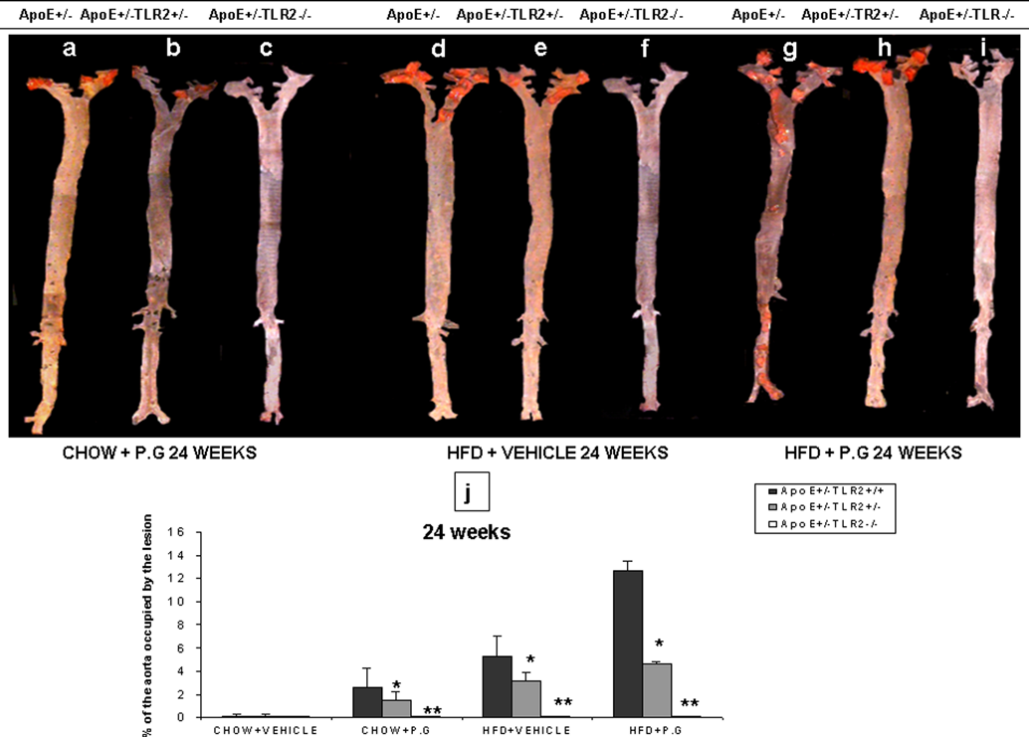
*P. g* and/or high fat diet increases aortic atherosclerotic lesions in ApoE^+^/^−^-TLR2^+/+^ mice when compared to ApoE^+/−^-TLR2^+/−^, and ApoE^+/−^-TLR2^−/−^ mice. *En face* analysis: (1A–1I): Representative *en face* view of aortic surface lesions in ApoE^+^/^−^-TLR2^+/+^, ApoE^+/−^-TLR2^+/−^, and ApoE^+/−^-TLR2^−/−^ mice after 24 weeks of treatments. (1J): Calculated percentages of aortic surface area covered by lesions after 24 weeks of treatments (bacterial challenge or vehicle control) among mice of three genotypes maintained on standard chow or high fat diets. Values represent means±SD; *p<0.05 for ApoE^+/−^-TLR2^+/+^ mice compared to ApoE^+/−^-TLR2^+/−^ mice and **p<0.05 for ApoE^+/−^-TLR2^+/+^ mice compared to ApoE^+/−^-TLR2^−/−^ mice in the same treatment condition and maintained on the same diet. Abbreviations are as defined in the text.

Quantitative *en face* analysis revealed statistically significant smaller lesions in ApoE^+/−^-TLR2^+/−^ mice compared to ApoE^+/−^-TLR2^+/+^ mice after 24 week treatments (p<0.05): High fat diet inoculated with *P. g* (HP) demonstrated 12.5±1.7%, High fat diet injected with vehicle (HS) 5.2±1.8% and Chow diet inoculated with *P. g* (CP) demonstrated 2.6±0.8% of the aorta occupied by lesion. Interestingly, we did not observe any lesions on the aortic surface in ApoE^+/−^-TLR2^−/−^ mice, irrespective of the diet or inoculum (Figs 1A–J). We also did not observe any lesions in any of the mice that were maintained on a chow diet and injected with vehicle (CS), regardless of their genetic backgrounds. We previously observed similar results from *en face* analysis performed after 14 weeks of these inoculation treatments of mice these same three genotypes (data not shown).

**Figure 2:**
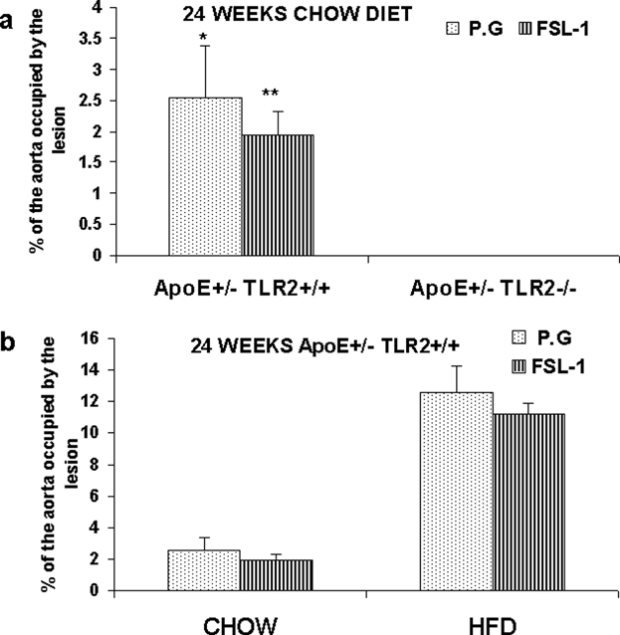
TLR2 activation through FSL-1 demonstrated no significant difference in aortic lesions when compared to *P. g* in ApoE^+/−^-TLR2^+/+^ and ApoE^+/−^-TLR2^−/−^ mice. (2A) Percentage of aortic surface area covered by lesions in chow-fed groups for mice from two genetic backgrounds (ApoE^+/−^-TLR2^+/+^ and ApoE^+/−^-TLR2^−/−^) injected with *P. g* or FSL-1 for 24 weeks. Values represent means±SD; *p<0.05 between ApoE^+/−^-TLR2^+/+^ mice and ApoE^+/−^-TLR2^−/−^ mice injected with *P. g*; **p<0.05 between ApoE^+/−^-TLR2^+/+^ mice and ApoE^+/−^-TLR2^−/−^ mice injected with FSL-1. No lesions were detected in ApoE^+/−^-TLR2^−/−^ mice irrespective of the treatment. (2B) Percentage of aortic surface area covered by lesions in ApoE^+/−^-TLR2^+/+^ mice maintained on either diet and injected weekly with *P. g* or FSL-1 for 24 weeks. Values represent means±SD.

Similarly, FSL-1 treatment for 24 weeks failed to induce any atherosclerotic changes in the aortas of ApoE^+/−^-TLR2^−/−^ mice maintained on standard lab chow (data not shown). However, in ApoE^+/−^-TLR2^+/+^ mice maintained on a high fat diet and treated with FSL-1 for 24 weeks,11.2±0.6% of the aorta was covered by lesion. In contrast, in mice maintained on standard chow, FSL-1 treatment for 24 weeks resulted in significantly smaller atherosclerotic lesions that occupied only 1.94%±0.39% of the aorta (Fig. 2A). No statistically significant differences could be observed in the extent of aortic lesions in ApoE^+/−^-TLR2^+/+^ mice injected with *P.g* or FSL-1, irrespective of the diet (Fig. 2B).

**Figure 3:**
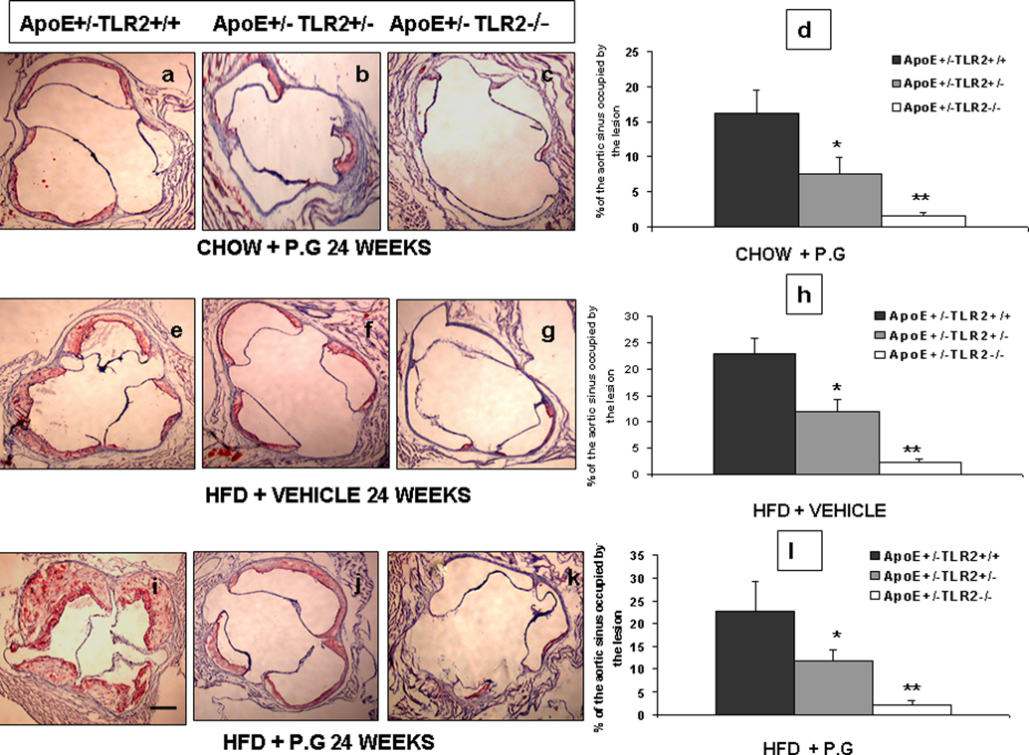
*P.g* and/or high fat diet increases atherosclerotic lesions in proximal aorta of ApoE^+^/^−^-TLR2^+/+^ mice when compared to ApoE^+/−^-TLR2^+/−^, and ApoE^+/−^-TLR2^−/−^ mice. Microscopic cross-sections (10 µm) of the proximal aortic root were stained with Sudan IV and counterstained with hematoxylin to reveal lipid deposition, which was quantified by digital morphometry. (3A–D): results from mice maintained on a standard chow diet and inoculated weekly with *P. g* (CP). (3E–H): results from mice maintained on a high fat diet and inoculated weekly with vehicle (HS) (normal saline). (3I–L) results from mice maintained on a high fat diet and inoculated weekly with *P. g* (HP). (3D, H, l): data are presented graphically as percentage of total lumen of the proximal aorta occupied by lesions after 24 weeks of injections. Values represent means±SD; *p<0.05 between ApoE^+/−^-TLR2^+/+^ mice and ApoE^+/−^-TLR2^+/−^ mice; **p<0.05 between ApoE^+/−^-TLR2^+/+^ mice and ApoE^+/−^-TLR2^−/−^ mice in the same condition and maintained on the same diet. Abbreviations are as defined in the text. Photomicrographs shown are representative images obtained at the end of the 24 week treatment period. Original magnifications 20×. Scale bar represents 0.5 mm.

**Figure 4:**
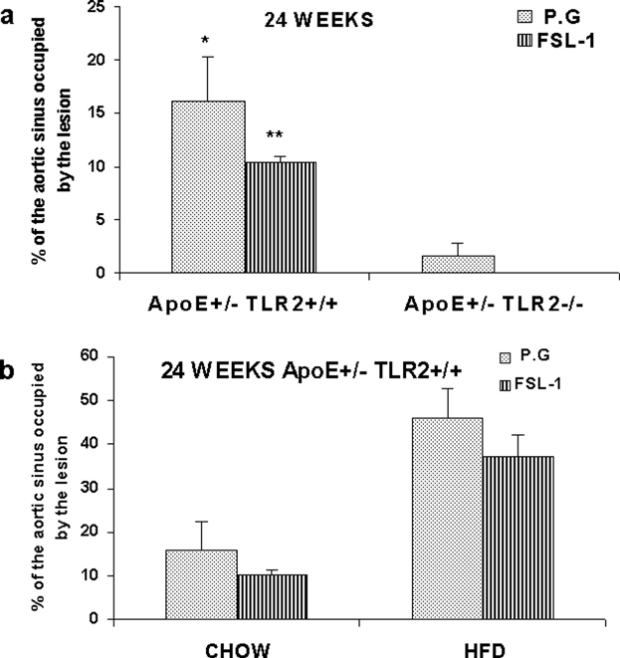
TLR2 activation through FSL-1 demonstrated no significant difference in proximal aortic lesions when compared to *P. g* in ApoE^+/−^-TLR2^+/+^ and ApoE^+/−^-TLR2^−/−^ mice. Microscopic cross-sections (10 µm) of the proximal aortic root were stained with Sudan IV and counterstained with hematoxylin to reveal lipid deposition, which was quantified by digital morphometry for samples from ApoE^+/−^-TLR2^+/+^ and ApoE^+/−^-TLR2^−/−^ mice. (4A): percentage of total lumen of the proximal aorta occupied by lesions after 24 weeks of treatment in ApoE^+/−^-TLR2^+/+^ and ApoE^+/−^-TLR2^−/−^ mice maintained on a standard chow diet and injected weekly with *P. g* or FSL-1. Values represent means±SD; *p<0.05 for differences between mice injected with *P. g*; **p<0.05 for differences between mice injected with FSL-1. No lesions were detected in ApoE^+/−^-TLR2^−/−^ mice irrespective of the treatment. (4B): percentage of total lumen of the proximal aorta occupied by lesions in ApoE^+/−^-TLR2^+/+^ mice maintained on a chow diet or a high fat diet after 24 weeks of injections with *P. g* or FSL-1. Values represent means±SD.

Histomorphometric analysis revealed significantly smaller lesions in the proximal aortas of ApoE^+/−^-TLR2^+/−^ mice compared to ApoE^+/−^-TLR2^+/+^ mice and ApoE^+/^-TLR2^−/−^ mice. In mice fed with high fat diet and injected with *P.g* the proximal aorta occupied by lesion was 46.2±6.6% in ApoE^+/−^-TLR2^+/+^, 18.6±2.4 in ApoE^+/−^-TLR2^+/−^ and 3.04±0.8 in ApoE^+/^-TLR2^−/−^ mice. In mice fed with high fat diet and injected with saline the proximal aorta occupied by lesion was 22.7±2.9% in ApoE^+/−^-TLR2^+/+^, 11.7±2.5 in ApoE^+/−^-TLR2^+/−^ and 2.2±0.6 in ApoE^+/^-TLR2^−/−^ mice. In mice fed with chow diet and injected with *P.g* the proximal aorta occupied by lesion was 16.1±3.2% in ApoE^+/−^-TLR2^+/+^, 7.6±2.3 in ApoE^+/−^-TLR2^+/−^ and 1.5±0.5 in ApoE^+/^-TLR2^−/−^ mice. (Fig. 3) Similar results were obtained at 14 weeks post inoculation also (data not shown). However, we did not observe any lesions in the chow-fed, vehicle-treated mice (CS), irrespective of their TLR2 genotype. Histomorphometric analysis of the proximal aortas revealed that 37.5±4.6% of the aortic lumen was occupied by lesion in ApoE^+/−^-TLR2^+/+^ mice injected with FSL-1 and maintained on a high fat diet, compared with only 10.4±1.3% of ApoE^+/−^-TLR2^+/+^ mice kept on a chow diet (Fig 4A). No lesions were observed in ApoE^+/−^-TLR2^−/−^ mice after 24 weeks of FSL-1 injections and a standard chow diet (data not shown). Furthermore, there was no statistically significant difference in the percentage of aortic lumen occupied by lesions in mice injected with *P. g* when compared to FSL-1 treatment, irrespective of diet (Fig 4B).

### Imunohistochemical Analysis of Proximal Aorta

**Figure 5:**
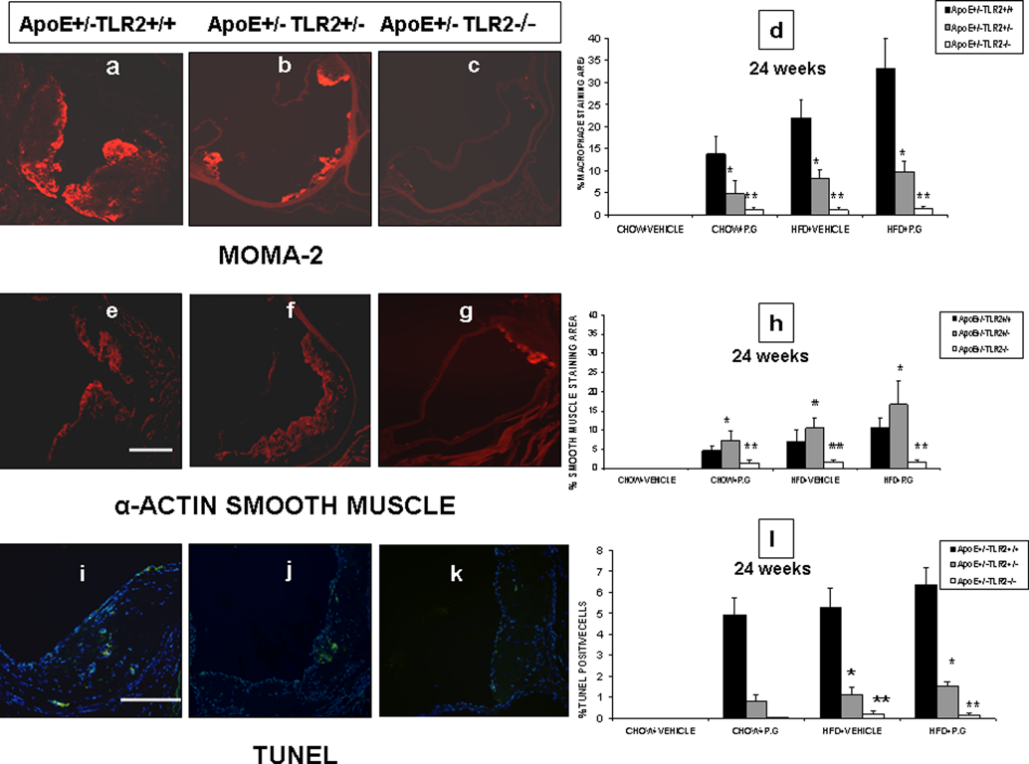
*P. g* and/or high fat diet results in unstable plaque in ApoE^+^/^−^-TLR2^+/+^ mice when compared to ApoE^+/−^-TLR2^+/−^, and ApoE^+/−^-TLR2^−/−^ mice. Representative photomicrographs of atherosclerotic plaques from the aortic sinus of ApoE^+/−^-TLR2^+/+^, ApoE^+/−^-TLR2^+/−^ and ApoE^+/^-TLR2^−/−^ mice maintained on a high fat diet and inoculated weekly with *P. g* (HP) for 24 weeks. Stains identify sections of macrophage infiltration (MOMA-2 red staining) (5a, 5b&5c); smooth muscle cells (α-SMA red staining) (5E, 5F&5G); TUNEL positive cells (green spots coinciding with nuclear stain DAPI) (5I, 5J&5K). Quantitative computer-assisted image analysis (as described in Materials and Methods) was used to quantify the percentage of macrophage-positive areas (5D), smooth muscle cell area (5H) and TUNEL/DAPI positive cells (5I) in proximal aortic lesions in ApoE^+/−^-TLR2^+/+^, ApoE^+/−^-TLR2^+/−^ and ApoE^+/^-TLR2^−/−^ mice of all the groups at the conclusion of the 24 week treatment period. Data represent means±SD; *p<0.05 for ApoE^+/−^-TLR2^+/+^ mice compared to ApoE^+/−^-TLR2^+/−^ mice, and **p<0.05 for ApoE^+/−^-TLR2^+/+^mice compared to ApoE^+/−^-TLR2^−/−^ mice in the same treatment condition and maintained on the same diet. Abbreviations are as defined in text. Original magnifications 100× for macrophages and smooth muscle and 200× for TUNEL/DAPI staining. Scale bar represents 0.5 mm.

After establishing the involvement of TLR2 in diet/bacteria induced atherosclerosis, we performed a detailed examination of plaque compositions by immunofluorescence staining. Five sections of the proximal aorta per animal (n = 8) each separated by 80 µm, were selected and staining specific for macrophages, smooth muscle cells, and apoptotic cells using MOMA-2, α-SMA and TUNEL were performed, respectively. Significant differences in the plaque composition were observed between atherosclerotic lesions of all ApoE^+/−^-TLR2^+/+^ mice when compared to those from ApoE^+/−^-TLR2^+/−^ and ApoE^+/−^-TLR2^−/−^ mice, irrespective of the treatment. The atherosclerotic lesions in ApoE^+/−^-TLR2^+/+^ mice exhibited a greater percentage of infiltrating macrophages than smooth muscle cell accumulation. In contrast, in ApoE^+/−^-TLR2^+/−^ and ApoE^+/−^-TLR2^−/−^ mice, macrophage content was either less than or equal to the smooth muscle cell accumulation in plaques (Figs. 5A–5G). Furthermore, the marked increase in the inflammatory component of the lesions in ApoE^+/−^-TLR2^+/+^mice was associated with a substantial increase in the occurrence of apoptosis within their plaques. In ApoE^+/^-TLR2^+/+^ mice we observed: 6.3%, 5.2% and 4.9% inflammatory component in the HP, HS and CP groups, respectively (Figs. 5I–L)

### Serum Amyloid A Level

**Figure 6:**
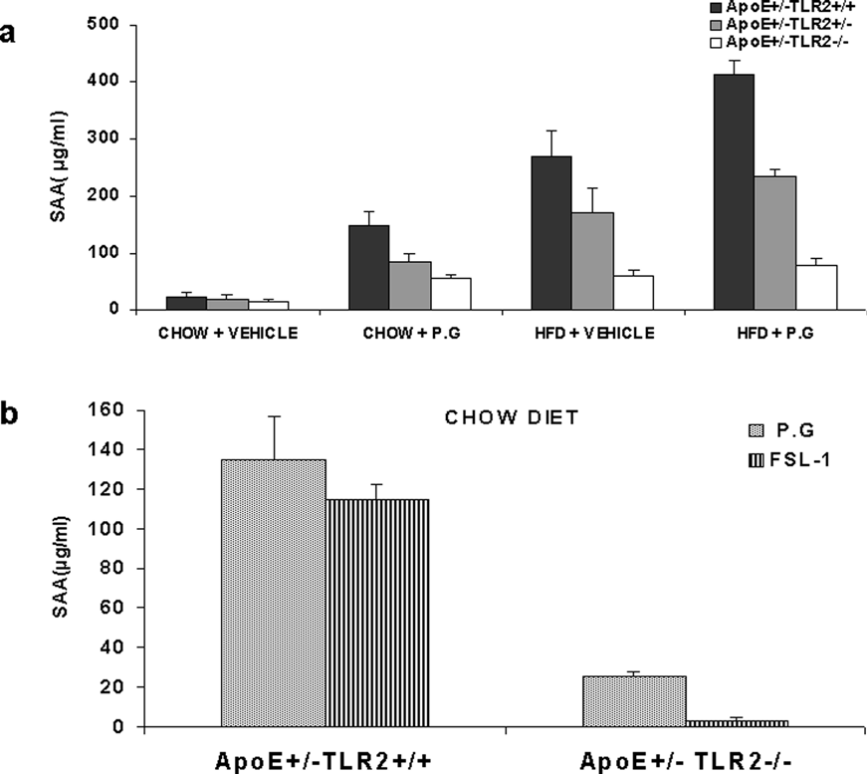
TLR2 activation through FSL-1 demonstrated no significant difference in SAA levels when compared to *P. g* in ApoE^+/−^-TLR2^+/+^ and ApoE^+/−^-TLR2^−/−^ mice. (6A) SAA levels in serum samples obtained after 24 weeks of inoculations, as determined by ELISA. Data represent means±SD; *p<0.05 between ApoE^+/−^-TLR2^+/+^mice and ApoE^+/−^-TLR2^+/−^ mice; **p<0.05 between ApoE^+/−^-TLR2^+/+^mice and ApoE^+/−^-TLR2^−/−^ mice maintained under the same conditions and on the same diet. Abbreviations are as defined in the text. (6B) SAA levels in serum samples obtained at the end of the study, determined by ELISA. Data represent means±SD in ApoE^+/−^-TLR2^+/+^ mice maintained on either standard chow or a high fat diet, and injected weekly with *P. g* or FSL-1.

At the conclusion of the 24 week treatment period, SAA levels were highest in mice on high fat diets and also inoculated with *P.g*. High fat mice inoculated only with saline had the next highest SAA levels, followed by mice on a normal diet but challenged with weekly inoculations of *P.g*. Mice from all genetic backgrounds maintained on standard chow and inoculated with vehicle had the lowest SAA levels, regardless of genotype (Fig 6A). The serum SAA levels were undetectable in ApoE^+/−^-TLR2^−/−^ mice maintained on a chow diet and injected with FSL-1 (data not shown). Also, there was no significant difference in serum SAA levels between ApoE^+/−^-TLR2^+/+^ mice injected with FSL-1 when compared to the *P. g* injected group, irrespective of the diet (Fig 6B).

### Serum Cytokine Levels

**Figure 7:**
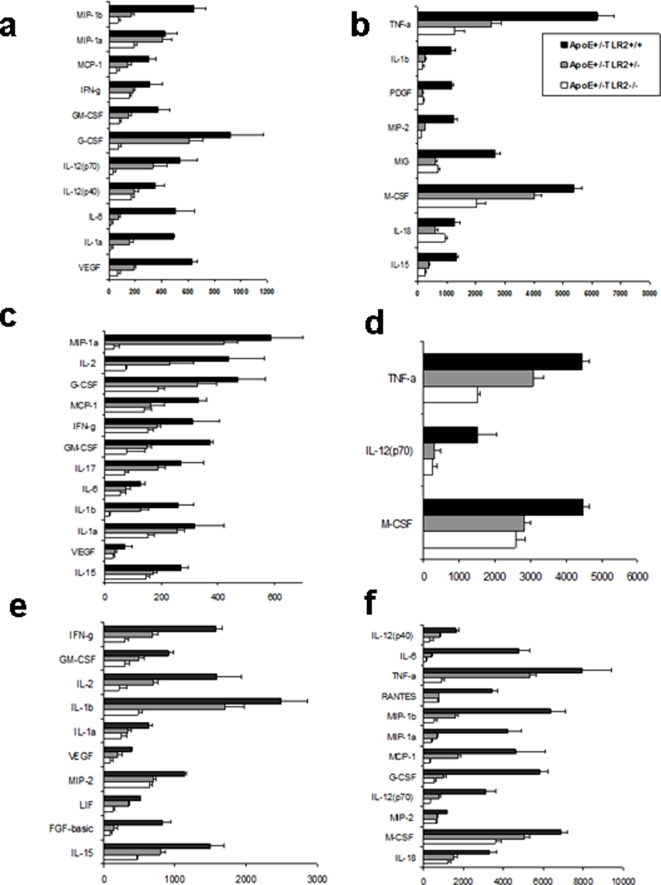
*P. g* and/or high fat diet results in increased proinflamatory cytokines in ApoE^+^/^−^-TLR2^+/+^ mice when compared to ApoE^+/−^-TLR2^+/−^, and ApoE^+/−^-TLR2^−/−^ mice. Serum cytokine levels (pg/ml) in mice maintained on a standard lab chow diet and inoculated weekly for 24 weeks with *P. g.* (Fig 7A&7B). Serum cytokine levels (pg/ml) in mice maintained on a high fat diet and injected with saline weekly for 24 weeks (Fig 7C&7D). Serum cytokine levels (pg/ml) in mice maintained on a high fat diet and inoculated weekly for 24 weeks with *P. g* (Fig 7E&7F).

To further correlate the serum cytokine levels within advanced stage atherosclerotic lesions, serum samples were analyzed for 32 cytokines. Statistical significance was evaluated by ANOVA followed by the post-hoc Scheffe test. A level of p<0.05 was considered significant. Significantly altered cytokine levels were observed as a consequence of the high fat diet and of inoculation with *P. g*. These are presented in supplement sheet Fig-3. Compared to ApoE^+/−^-TLR2^+/−^ and ApoE^+/−^-TLR2^−/−^ mice, ApoE^+/−^-TLR2^+/+^ mice displayed profoundly higher levels of most proinflammatory cytokines and chemokines, including IL-1α, IL-1β, IL-6, IL-12p40, IL-12p70, TNF-α, MCP-1, VEGF, M-CSF, and GM-CSF in high fat diet and/or bacterial-challenged animals. No significant differences were observed in the cytokine levels between all 3 genetic background mice fed the chow diet and saline-inoculated. The most significantly elevated cytokine levels were seen in ApoE^+/−^-TLR2^+/+^ mice fed the high fat diet and also inoculated with *P.g* (HP) (Fig. 7).

Cytokine profiling in serum samples obtained from ApoE^+/−^-TLR2^+/+^ mice treated for 24 weeks with FSL-1 or wild type *P.g.* were compared. The expression of cytokines was increased with both stimuli in ApoE^+/−^-TLR2^+/+^ stimulated with FSL-1 or wild-type *P.g.* irrespective of the diet (Fig. 3 and 4 Supplemental Data S1). FSL-1 did not stimulate cytokine expression in ApoE^+/−^-TLR2^−/−^ mice (Fig. S3 and S4 Supplemental Data S1).

### Two-dimensional Protein Maps of Aortic Tissues from ApoE^+/−^-TLR2^+/+^ and ApoE^+/−^-TLR2^−/−^ Mice

**Figure 8:**
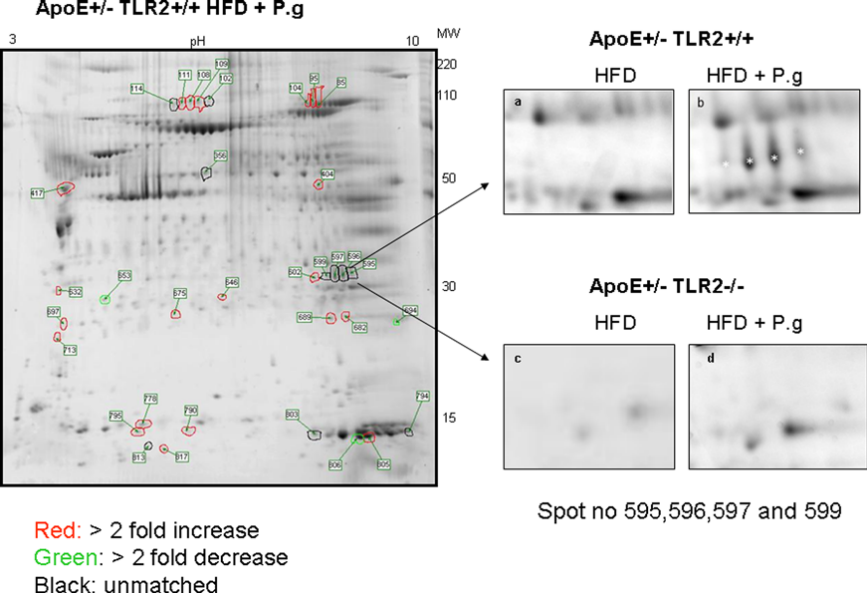
*P.g* and/or high fat diet demonstrated changes in the aortic protein in ApoE^+^/^−^-TLR2^+/+^ mice when compared to ApoE^+/−^-TLR2^−/−^ mice. Two-dimensional electrophoresis gel image of the proteins extracted from aortas (n = 5) from ApoE^+/−^-TLR2^+/+^ mice fed a high fat diet and injected weekly with *P. g*. Enlarged spots representing Hb were observed in aortas proteins from ApoE^+/−^-TLR2^+/+^ mice maintained on a high fat diet and injected with *P. g* (8B). Corresponding gel regions from aorta proteins from ApoE^+/−^-TLR2^+/+^ mice maintained on a high fat diet alone (8A) or ApoE^+/−^-TLR2^−/−^ mice fed a high fat diet alone (8C) or also injected with *P.g*. (8D) did not exhibit the spots. Gels were stained by SYPRO RUBY stain. The spot numbers correspond to proteins listed in Table 1.

Protein extracts from aortic tissues of ApoE^+/−^-TLR2^+/+^ and ApoE^+/−^-TLR2^−/−^ mice maintained on a high fat diet and/or inoculated with *P. g* were separated using 2-DGE. An example of the overall 2-DGE patterns of aortic protein extracts of ApoE^+/−^-TLR2^+/+^ mice fed a high fat diet and inoculated with *P. g* is shown in Fig 8. A total of 34 different protein spots were detected in response to *P.g* challenge: 21 protein spots increased least by 2-fold (Fig 8 red); 3 protein spot decreased at least by 2-fold (Fig 8 green) and 10 unmatched protein spots (Fig 8 black). Out of the 21 protein spots with at least 2 fold increase 6 proteins: Vesl-2 protein, Sod-2 protein, fumarate hydratase, myosin light chain polypeptide 3, aconitase, and gelsolin were identified (Table 1). Out of the 10 unmatched spots a protein: Hb was identified (table 1) only in ApoE^+/−^-TLR2^+/+^ mice maintained on the high fat diet and inoculated with *P. g*. Magnified gel regions corresponding to Hb from mice of both genotypes maintained on high fat diets after challenge with *P.g* or vehicle are compared in Figs 8A–D.

**Table 1:** 1 Protein identified by MALDI TOF.

Spot number	Protein	Expectation	Accession no.	ApoE+/− TLR2+/+
				*HFD+P.g*
111,108,109	Gelsolin	5.30E-05	8606238	>2fold increase
104,95,80	Aconitase	1.20E-07	18079339	>2 fold increase
599,597,596,595	Hemoglobin	8.30E-06	31982300	Unmatched
689	SOD-2	6.05E-06	17390379	>2 fold increase
682	Vesl-2	2.00E-04	3766297	>2 fold increase
404	Fumarate hydratase	1.30E-05	33859554	>2 fold increase
417	Myosin, light polypeptide 3	5.70E-04	33563264	>2 fold increase

Identification of proteins differentially expressed in ApoE^+/−^-TLR2^+/+^ mice fed a high fat diet and inoculated with *P. g* as compared to other groups.

## Discussion

In the present study we demonstrate that TLR2 plays an important role in the pathogenesis of bacteria-enhanced diet-dependent atherosclerosis in the ApoE^+/−^ murine model, establishing a key link between atherosclerosis and immune defense against foreign pathogens and/or endogenous inflammatory ligands. Both *en face* and histomorphometric data revealed that a greater percentage of the aorta and aortic lumen was occupied by the atherosclerotic lesions in ApoE^+/−^-TLR2^+/+^ mice as compared to either ApoE^+/−^-TLR2^+/−^ or ApoE^+/−^-TLR2^−/−^ mice. ApoE^+/−^-TLR2^+/+^ mice fed a high fat diet and inoculated with *P. g* (HP) exhibited larger lesions compared to mice fed a high fat diet and inoculated only with saline vehicle (HS) or mice fed the standard lab chow diet and inoculated with *P. g*. (CP). Increased cholesterol, LDL and decreased HDL level seen in ApoE^+/−^-TLR2^+/+^ mice injected with *P. g* in current work, corroborates well previous studies showing that periodontal pathogens can influence the systemic lipoprotein profile [33].

Our data support previous studies showing that both endogenous (diet) and exogenous (Pam3CSK4) TLR2 ligands play important roles in the modulation of atherosclerosis [14], [28]. Indeed, mice deficient in TLR4, TLR2 and MyD88 all have reduced atherosclerosis which establishes that TLR-dependent pathways contribute to disease development. Although it is likely that total “infectious burden” contributes to atherosclerosis progression, endogenous ligands may also initiate and modulate Toll-like receptor signaling pathways [29], [30], [31].

The unstable plaque phenotype is characterized by increased vulnerability to rupture and thrombosis. Histologically, an unstable plaque is identified by its thin fibrous cap, low smooth muscle cell count, high macrophage content, increased apoptosis and large lipid core [32], [33], [34], [35], [36]. The loss of the smooth muscle cells in particular is thought to be detrimental for plaque stability since most of the interstitial collagen fibers, which are important for the tensile strength of the fibrous cap, are produced by these cells [37]. Our detailed immunohistochemical analysis of atherosclerotic lesions for smooth muscle cells, macrophages, and apoptotic regions found that all the signs of plaque instability were consistently observed in ApoE^+/−^-TLR2^+/+^ mice, while ApoE^+/−^-TLR2^+/−^ and ApoE^+/−^-TLR2^−/−^ mice showed lesions more characteristic of stable plaque, with less macrophage content, less apoptosis, smaller lipid cores, and higher smooth muscle cell mass (Fig. 5). A significant increase of apoptosis in the lesions of ApoE^+/−^-TLR2^+/+^ mice, along with upregulation of proinflammatory cytokines which regulate the release of the matrix-degrading proteinases and may favor the unstable plaque phenotype [38].

SAA, the mouse counterpart of human C-reactive protein, is an acute phase reactant known as a marker for systemic inflammation. It has been demonstrated that CRP is produced in the liver in response to IL-6, IL-1β, and TNF-α [39]. A strong association has also been shown between circulating levels of SAA and the extent of atherosclerosis in the aorta [23], [26], [27]. Our data demonstrated significantly higher serum levels of SAA, IL-6, IL-1β, and TNF-α in ApoE^+/−^-TLR2^+/+^ mice compared with ApoE^+/−^-TLR2^−/−^ mice, irrespective of the diet or bacterial challenge treatment. Furthermore, SAA levels significantly correlated with the extent of aortic lesions examined after 24 weeks of challenge. Interestingly, significantly lower levels of SAA in ApoE^+/−^-TLR2^−/−^ mice suggest that TLR2 deficiency may also lead to a lower overall systemic inflammatory status.

We performed cytokine profiling in order to further investigate the systemic inflammatory status associated with TLR2 deficiency and the involvement of TLR2 in atherosclerosis. Our data show that TLR2 elicits differential expression of inflammatory cytokines and co-stimulatory molecules upon challenge with atherogenic stimuli (*P.g.* and/or high fat diet). Maximum induction of a host of proinflammatory cytokines (IL-1α, IL-1β, IL-6, IL-18, IFN-γ, IL-12p40, IL-12p70, TNF-α, MCP-1, VEGF, M-CSF, and GM-CSF) was observed in ApoE^+/−^-TLR2^+/+^ mice maintained on a high fat diet and challenged with *P. g.* Most of these cytokines and chemokines are proinflammatory factors, favoring cell migration, proliferation [40], [41] and chemo-attraction of inflammatory cells, such as monocytes/macrophages and T cells [42], [43], [44]. These results further implicate an aspect of antigen-specific adaptive immunity mostly characteristic of a Th1 response, including cytokines IL-2, IL-18, IFNγ and TNF-α [9]. The differential cytokine induction also implies that *P. g* and/or a high fat diet can activate different receptors to mediate intracellular signaling. It is known that the formation of heterodimers between TLR2 and other TLRs (TLR1 or TLR6) dictates the specificity of ligand recognition, thereby diversifying the possible outcomes of TLR2 activation [20]. In this context, it is noteworthy that the ApoE^+/−^-TLR2^−/−^ genotype conferred atheroprotective effects, which may result in part from reduced systemic inflammation as shown by reduced expression of proinflammatory cytokines and chemokines in all treatment groups.

To further establish the role of TLR2 in modulating the progression of atherosclerosis, we stimulated mice with the TLR2 agonist known as FSL-1. *En face* and histomorphometric analysis revealed that systemic exposure to FSL-1 dramatically increased lesion severity in a manner similar to *P. g*. In contrast, the absence of TLR2 resulted in complete prevention of lesions in mice on the chow diet and injected with FSL-1. Furthermore, the percentage of the lesions observed both in aorta and aortic sinus were comparable to the lesions observed in ApoE^+/−^-TLR2^+/+^ mice injected with *P. g* irrespective of the diet after 24 weeks. FSL-1 stimulation also altered the systemic inflammatory status as monitored by increased serum SAA levels in the ApoE^+/−^-TLR2^+/+^ mice when compared to the ApoE^+/−^-TLR2^−/−^ mice. It is noteworthy that the levels of serum SAA in ApoE^+/−^-TLR2^+/+^ mice stimulated with FSL-1 were comparable to the levels obtained when mice were challenged with *P. g* irrespective of the diet, thus confirming the role of TLR2 in upregulation of systemic inflammation produced by *P. g* challenge. Cytokine profiling showed that both FSL-1 stimulation and *P.g* challenge resulted in a relatively similar expression of proinflammatory cytokines.

Our expression proteomic approach extends the growing body of literature linking TLR2 and atherogenesis by identifying proteins involved in *P. g-* and/or diet-induced atherosclerosis in ApoE^+/−^ mice. Using 2D gel electrophoresis (2-DGE) in combination with mass spectrometry (MS), we found that in ApoE^+/−^-TLR2^+/+^ mice, *P. g* stimulation in combination with a high fat diet up-regulated the expression of a set of proteins (Hb, Vesl-2 protein, Sod-2 protein, fumarate hydratase, myosin light chain polypeptide 3, aconitase and gelsolin) compared to high fat diet alone. Some of these proteins were found of interest in improving our understanding of the mechanisms linking atherogenesis to infection, inflammation, and immune response.

Hemoglobin (Hb) is known to enhance the biological function of bacterial endotoxins [48] and therefore increased Hb can contribute to aheightened systemic response. Furthermore increased Hb content in the blood leads to increased viscosity, with detrimental effects on blood flow. Moreover, intraplaque hemorrhage seen in advanced lesions also causes the deposition of Hb. In our study, the detection of Hb only in ApoE^+/−^-TLR2^+/+^ mice fed a high fat diet and injected with *P. g* indicates that elevated Hb levels may be useful as a biomarker for an unstable plaque phenotype. Its presumed mechanism of vascular injury would include both oxidative heme toxicity caused by its ineffective clearance and also the subsequent consumption of nitric oxide, an important mediator of vascular homeostasis.

Vesl-2 (Homer 2) is a post-synaptic adaptor protein that has been shown to be present in multiple tissues such as brain and heart. It may be linked to atherogenesis through its associations with glutamate receptor complexes and also the actin cytoskeleton. These glutamate receptors are coupled with G-proteins and activate phospholipase C, ultimately activating the IP3 receptor (IP3R) to release intracellular calcium, which can alter the function of ECs. Endothelial dysfunction typically results in platelet aggregation at the damaged site. Elevated intracellular calcium also leads to increased uptake of macromolecules in plasma such as fibrinogen and LDL, eventually forming atherosclerotic plaque. Thus it may be speculated that increased vesl-2 protein in ApoE^+/−^-TLR2^+/+^ mice fed a high fat diet and injected with *P. g* may lead to an increase in intracellular calcium, contributing to the increased atherosclerosis in this group of mice.

Our observation of increased SOD-2 along with reduced smooth muscle cell content in ApoE^+/−^-TLR2^+/+^ mice fed a high fat diet and injected with *P. g* agrees well with a recent report in which SOD-2 deficient smooth muscle cells can exhibit a hypertrophic and hyperplastic phenotype. Thus we may speculate that *P.g* challenge upregulates mitochondrial SOD-2 and affects downstream pathways involving MAP kinases. Thus, increased SOD-2 may play a crucial role in determining plaque phenotype as it directly affects smooth muscle cell phenotype.

Gelsolin is an actin-binding protein that is a key regulator of actin filament assembly and disassembly. It is regulated by Ca^2+^- and polyphosphoinositide 4, 5-bisphosphate (PIP_2_) and plays an important role in actin remodeling by regulating actin filament severing and capping. It has also been shown to play a role in apoptosis [45] and in sepsis-induced cell injury. Increased gelsolin activity has also been shown in failing human hearts [46] probably in reaction to the cell injury. Thus, increased level of gelsolin seen in ApoE^+/−^-TLR2^+/+^ mice fed a high fat diet and injected with *P. g* may be linked to the increased apoptosis and atherosclerosis observed in this group.

Aconitase and fumarate hydratase are Krebs cycle enzymes. These enzymes area lso known to play important roles in the response to oxidant stress, which can inactivate aconitase and other Krebs cycle enzymes [47]. We observed increased aconitase and fumarate hydratase levels in ApoE^+/−^-TLR2^+/+^ mice fed a high fat diet and injected with *P. g.* These proteins probably represent an adaptive response to increased oxidative stress but they are also important predisposing factors in the progression of the atherosclerotic process.

Taken together, our results confirm the important role for TLR2 signaling in diet and/or bacteria enhanced atherosclerosis in an ApoE^+/−^ mouse model, providing a link between innate immunity, inflammation and atherosclerosis. Due to TLR2 central role in the disease process, it represent a target of immunomodulatory therapy with the goal of tipping the balance from excessive chronic inflammation towards resolution of inflammation, while not compromising host defenses or atheroprotective immune functions. Therefore manipulation of TLR2 pathways has great therapeutic potential. TLRs inhibitors or their associated signaling molecules hold great promise in the prevention of atherosclerosis.

## Materials and Methods

Please refer to the supplemental data and Fig. S1 and S2 for details. Briefly, all animal protocols were approved by the Boston University Medical Campus Institutional Animal Care and Use Committee. To investigate the role of TLR2 in inflammation- and/or infection-associated atherosclerosis, 10 week-old ApoE^+/−^-TLR2^+/+^, ApoE^+/−^-TLR2^+/−^ and ApoE^+/−^-TLR2^−/−^ mice were fed either a high fat diet or a regular chow diet. All mice were inoculated intravenously, once per week for 24 consecutive weeks, with 50 µl live *P. g* (10^7^ CFU) or vehicle (normal saline). Animals were euthanized 24 weeks after the first inoculation. Histomorphometric analysis of the aortic lesions and the proximal aorta using Sudan red stain were performed. Immunofluroscent staining for macrophage, smooth muscle cell and apoptosis were performed on the proximal aortic sections. Metabolic profile, serum amyloid A and serum cytokine levels were also performed for all the three genotypes. For the TLR2 agonist study a second set of four week old male ApoE^+/−^-TLR2^+/+^ fed either a HFD or a regular chow diet for 6 weeks (n = 10) were used. All mice were inoculated intravenously, once per week for 24 consecutive weeks, with 5 µg FSL-1in 50 µl saline or vehicle (normal saline). Animals were euthanized 24 weeks after the first inoculation. To compare and further establish an absolute effect of TLR2 in bacteria-enhanced atherosclerotic lesions, a third set of experiments involved only ApoE^+/−^-TLR2^−/−^ mice fed only the standard chow diet. Four week old ApoE^+/−^-TLR2^−/−^ mice maintained on a regular chow diet for 6 weeks (n = 10), were inoculated with 50 µl saline vehicle or 5 µg FSL-1 in 50 µl saline. All groups were analyzed for atherosclerotic lesions, metabolic profile, serum amyloid A and serum cytokine levels after 24 weeks of inoculations.

### Statistical Analysis

All histomorphometric measurements were made by an examiner blinded to the identity of the samples. All quantitative measurements were confirmed by random analysis of one fourth of the specimens by the same examiner (R>0.92) and by another independent examiner (a pathologist) to ensure consistency. The intra-examiner and inter-examiner variation were each <10%. All histomorphometric and serum assay data were analyzed by ANOVA followed by the post-hoc Scheffe test. A level of p<0.05 was considered significant.

## Supporting Information

Data S1 Supplemental material document
(0.08 MB DOC)

Figure S1 Animal grouping and experimental time schedule for P.gingivalis expeiments. Four week old male ApoE+/−-TLR2+/+, ApoE+/−-TLR2+/− and ApoE+/−-TLR2−/− mice were fed either a HFD or a regular chow diet for 6 weeks (n = 8), then inoculated once per week for 24 weeks with 50 µl of either vehicle (normal saline) or 10^7^ CFU) P. g while maintained on the chosen diet. Thus, there were 4 groups for each genotype of mice: Group 1 was fed a standard chow diet and inoculated weekly with 50 µl saline vehicle (CS); Group 2 was fed a standard chow diet and inoculated with 50 µl (10^7^ CFU) P. g. (CP); Group 3 was fed a high fat diet and inoculated with 50 µl saline vehicle (HS); Group 4 was fed a high fat diet and inoculated with 50 µl (10^7^ CFU) P. g (HP). In summary, mice (n = 8) in each group received 24 tail vein injections of either vehicle or P. g once weekly.
(0.83 MB TIF)

Figure S2 Animal grouping and experimental time schedule for FSL-1expeiments. Effects of FSL-1 were tested in two sets of experiments. In the first, four week old male ApoE+/−-TLR2+/+ were fed either a HFD or a regular chow diet for 6 weeks (n = 10) then inoculated once per week for 24 weeks with 50 µl of either vehicle (normal saline) or 5 µg FSL-1 while maintained on the chosen diet. The resulting 4 groups were: Group 1a was fed a standard chow diet and inoculated with 50 µl saline vehicle (CS); Group 2a was fed a standard chow diet and inoculated weekly with 50 µl (5 µg) FSL-1; Group 3a was fed a high fat diet and inoculated weekly with 50 µl saline vehicle (HS); Group 4a was fed a high fat diet and inoculated with 50 µl (5 µg) FSL-1. All groups were tested after 24 weeks of their diet and inoculation regimens. For the second set of experiments, four week old ApoE+/−-TLR2−/− mice maintained on only a regular chow diet for 6 weeks (n = 10), then were divided into 2 groups: Group 1b was inoculated weekly with 50 µl vehicle saline (CS); Group 2b was inoculated weekly with 50 µl (5 µg) FSL-1. All groups were tested after 24 weeks of inoculations.
(0.53 MB TIF)

Figure S3 TLR2 activation through FSL-1 demonstrated similar expression of increased proinflamatory cytokines as compared chow fed and injected with P. g in ApoE+/−-TLR2+/+ mice. Serum cytokine levels (pg/ml) in ApoE+/−-TLR2+/+ mice fed a standard chow diet and injected weekly with P. g or FSL-1. Data represent mean+SD.
(0.78 MB TIF)

Figure S4 TLR2 activation through FSL-1 demonstrated similar expression of increased proinflamatory cytokines as compared high fat fed and injected with P. g in ApoE+/−-TLR2+/+ mice. Serum cytokine levels (pg/ml) in ApoE+/−-TLR2+/+ mice fed a high fat diet and injected weekly with P. g or FSL-1. Data represent mean+SD.
(0.77 MB TIF)

Table S1 Metabolic profiles of ApoE+/−-TLR2+/+, ApoE+/−-TLR2+/− and ApoE+/−-TLR2−/− mice maintained on a standard lab chow diet or a high fat diet, and injected weekly with either saline or with P. g, at 24 weeks. *Significance between ApoE+/−-TLR2+/+, ApoE+/−-TLR2+/− and ApoE+/−-TLR2−/− for respective groups. Abbreviations are as defined in the text.
(0.07 MB DOC)

Table S2 Metabolic profile of ApoE+/−-TLR2+/+ and ApoE+/−-TLR2−/− mice fed with either a standard lab chow diet or a high fat diet, and injected weekly with either P. g or FSL-1; measurements were obtained after 24 weeks of treatments.
(0.04 MB DOC)
