# Supplementary material for: Toll-Like Receptor-2 Mediates Diet and/or Pathogen Associated Atherosclerosis: Proteomic Findings
Source: PLoS One. 2008 Sep 12;3(9):e3204. doi: 10.1371/journal.pone.0003204 (PMC2527517; doi:10.1371/journal.pone.0003204)
Supplement: Data S1 — Supplemental material document (0.08 MB DOC) [file pone.0003204.s001.doc]

**Data S1**

**METHODS**

**Mice and Diets:** The Institutional Animal Care and Use Committee of Boston University approved all animal protocols. Founder mice of the TLR-2^−/−^ strain, and the corresponding control mice (C57BL/6J) were obtained from S. Akira (Osaka University, Osaka, Japan). Apolipoprotein E deficient homozygote (ApoE^-/-^) mice were obtained from Jackson Laboratories (Bar Harbor, ME) as breeding mice. Three mouse genotypes were generated: ApoE^+/-^-TLR2^+/+^, ApoE^+/-^-TLR2^+/-^, ApoE^+/-^-TLR2^-/-^ . Female ApoE^-/-^ and ApoE wild-type C57BL/6J males were bred to generate ApoE-deficient heterozygotes harboring wildtype TLR2 expression (ApoE^+/-^-TLR2^+/+^). ApoE^+/-^- TLR2^+/-^ mice were generated by crossing male ApoE^-/-^ with female TLR2^-/-^ mice. To obtain ApoE^+/-^-TLR2^-/-^ male ApoE^+/-^-TLR2^+/-^ mice were backcrossed with female TLR2^-/-^ mice. The genotype of all the strains was verified by the polymerase chain reaction method (genotyping protocols from Jackson Laboratory). The experimental protocols are shown in Fig. 1. Mice from the three experimental strains, ApoE^+/-^-TLR2^+/+^, ApoE^+/-^-TLR2^+/-^ and ApoE^+/-^-TLR2^-/-^, were weaned at 4 weeks of age, and randomly assigned to either a cholate-free high fat diet diet (HFD) (D12492 from Research Diet) or regular mouse chow (lab diet) containing 0.02% cholesterol and 4.5% fat (rodent diet 5001).Estrogen has been suggested to be cardioprotective in females [1] , therefore in this study only male mice from the three experimental strains, ApoE^+/-^-TLR2^+/+^, ApoE^+/-^-TLR2^+/-^ and ApoE^+/-^-TLR2^-/-^ were used.

**Bacterial Strain, Dose and Route of Inoculation**: *P. g* strain 381, a human isolate, was grown on anaerobic agar plates as described previously [2]. We used live bacterial inoculation (10^7^ CFU) because in preliminary experiments, heat-killed *P. g* failed to produce atherosclerosis. The dose (10^7^ CFU) and delivery route (intravenous) of the pathogen compares well with bacteremia encountered in humans following dental infection, periodontal surgery, scaling, tooth extraction, or flossing [3,4,5].

**Animal Grouping and Experimental Time Schedule**

1. **Experiments involving *P. gingivalis*:** Mice heterozygous for apolipoprotein E (ApoE^+/-^) are susceptible to atherosclerosis if maintained on a high fat diet, but not develop the disease spontaneously [6,7,8]. Four week old male ApoE^+/-^-TLR2^+/+^, ApoE^+/-^-TLR2^+/-^ and ApoE ^+/-^-TLR2^-/-^ mice were fed either a HFD or a regular chow diet for 6 weeks (n=8), then divided into 4 groups: Group 1 continued on a standard chow diet and received weekly inoculations of 50µl saline vehicle; these mice and are referred to as chow saline group (CS).. Group 2 mice continued on a standard chow diet and were inoculated weekly with 50µl (10^7^CFU) *P. g;* they are referred to as chow *P.g* group (CP). Group 3 mice continued on a high fat diet and received weekly inoculations of 50µl saline vehicle; these mice are referred to as high fat diet saline group (HS). Group 4 mice were maintained on a high fat diet and inoculated weekly with 50µl (10^7^CFU) *P. g;* they are referred to as high fat diet *P.g* group (HP)(Fig 1). In summary, mice (n=8) from each of 12 groups received 24 weekly tail vein injections of either vehicle or *P. g*.
2. **Experiments involving TLR2 Agonist (FSL-1):** A second set of four week old male ApoE^+/-^-TLR2^+/+^ were fed either a HFD or a regular chow diet for 6 weeks (n=10) and then divided into 4 groups for injections: Group 1a was maintained on the same standard chow diet and inoculated weekly with 50µl saline vehicle (CS). Group 2a was continued on the standard chow diet and inoculated weekly with 5µg FSL-1in 50µl saline; these mice are referred to as chow FSL-1. Group 3a was maintained on the high fat diet and inoculated weekly with 50µl saline vehicle; they are referred to as high fat diet saline group (HS). Group 4a was fed a high fat diet and inoculated weekly with 5µg FSL-1 in 50µl of saline and are referred to as high fat diet FSL-1 group. All groups were evaluated after 24 weeks of inoculations for atherosclerotic lesions, metabolic profile, levels of serum amyloid A and serum cytokines, and protein expression by proteomic analysis in this study (Fig. 2). To compare and further establish an absolute effect of TLR2 in bacteria-enhanced atherosclerotic lesions, a third set of experiments focused on only ApoE^+/-^-TLR2^-/-^ mice fed only the standard chow diet. Four week old ApoE^+/-^-TLR2^-/-^ mice were maintained on a regular chow diet for 6 weeks (n=10) , then divided into 2 groups: Group 1b was inoculated with 50µl saline vehicle, and are referred to as chow diet saline group (CS). Group 2b was inoculated with 5µg FSL-1 in 50µl saline, and are referred to as chow diet FSL-1 group. All groups were analyzed for atherosclerotic lesions, metabolic profile, serum amyloid A and serum cytokine levels after 24 weeks of inoculations.

**Tissue Harvesting and Preparation:** At then conclusion of the study period, and after overnight fasting, mice were heavily sedated with inhaled isoflurane (SOLVAY) and exsanguinated from the femoral arteries. The heart and aorta were perfused abundantly to avoid any blood contaminant then separated close to the heart and processed for histology and morphometry as described previously [9,10].

**Morphometric Analysis:** *En face* morphometric analysis of the aortic tree: The *en face* quantification of atherosclerosis in the aortic tree was determined by image analysis as described previously [9,10]. Briefly, the aortic tree was rinsed in 70% ethanol, followed by staining with a solution containing 0.5% Sudan IV (Sigma), 35% ethanol and 50% acetone for 6 minutes, followed by destaining in 80% ethanol for 5 minutes. Data are presented as the percentage of the aorta occupied by the lesions.

Histomorphometric and histopathological analysis of atheroma lesions in the proximal aorta: Proximal aortic cross-sections were prepared for quantitative and histopathological evaluation of atherosclerotic lesions as described previously [11]. Briefly stated, five sections per animal, each separated by 80 µm, were stained (Sudan IV), counterstained (hematoxylin), and assessed with a computer-assisted image analysis program (Image ProPlus 4.0). The total cross-sectional area of the aortic lumen and lesion from the five images were measured.. The values were averaged and then the percentage lesion was expressed as a ratio of total lesion size / total sinus area.

**Immunohistochemistry:** To further determine the plaque phenotype, five sections of the proximal aorta per animal (n=8), each separated by 80 µm, were fixed with ice cold acetone for 10 minutes. After incubation with blocking buffer for an hour, sections were incubated with either a primary antibody against a monocyte/macrophage marker (MOMA-2; 1:25) (Chemicon) or an antibody against alpha smooth muscle actin (α-SMA;1:50) (Sigma) for one hour. Detection was accomplished using biotinylated secondary antibody and Texas red-conjugated avidin (Vector labs). Nonimmune IgG from appropriate species were used as negative controls. Stained macrophages or smooth muscle cells within atherosclerotic plaques in the aortic sinus were quantified using Image ProPlus 4.0. The ratio of the macrophage or smooth muscle stained area to total plaque area was calculated and plotted as described previously [12]. Apoptosis was detected by TUNEL staining of five frozen sections per animal, each separated by 80 µm, using a DeadEnd fluorimetric kit (Promega) according to the manufacturer’s instructions. Data are expressed as a percentage of positive cells relative to the total number of cells in the field of the lesion examined [12]. Samples processed in the absence of the TdT enzyme were used as negative controls. The specificity of the TUNEL signal was confirmed by switching the filters to DAPI excitation wavelength and confirming that TUNEL (FITC) signal co-localized with DAPI (nuclear stain).

**Lipids and Glucose Analysis:** Serum samples from all groups of mice (n=8/group) were evaluated for total cholesterol (TC; fluorometric assay based kit; Cayman Chemicals), HDL, LDL (colorimetric assay based kit;Wako Chemicals) and glucose levels (colorimetric assay based kit; BioAssay Systems). Each sample was analyzed three times in triplicate according to the kit manufacturer’s instructions.

**ELISA:** Venous blood was collected from mice at the time of euthanasia. After clotting at room temperature, blood samples were centrifuged for 30 minutes at 2000 *x* *g*, and the supernatants were collected and stored at -80°C. SAA levels were determined by ELISA (Biosource International) following the manufacturer’s instructions.

**Multianalyte Microbead Array to Detect Cytokine Expression:** Serum samples from mice euthanized after 24 weeks (n=8/group) were analyzed for inflammatory mediators using a mouse 23-plex and 9-plex cytokine microbead suspension array system (Bio-Plex; Bio-Rad, Hercules, CA). This microbead assay allows for the simultaneous detection of 32 individual inflammatory molecules. These assays were performed three times in triplicates following manufacturer’s instructions.

**Protein Sample Preparation:** After treatment had been completed, protein was extracted from the aortas of the ApoE^+/-^-TLR2^+/+^ and ApoE^-/-^-TLR2^-/-^ mice (n=5 in each group). This was accomplished in two steps. First, aorta tissues were cut into small pieces (5mm) and ground in a liquid nitrogen-cooled mortar and homogenized, followed by the addition of 1 ml of extraction/lysis buffer, per tissue extract (Bio-Rad total protein extraction kit lysis buffer, plus 10µl Bio-Lytes and 20 µl protease inhibitor cocktail per sample). Samples were held on ice and subjected to sonication for five 10-second pulses, then centrifuged at 13,000x *g* for 30 minutes at 4° C. The supernatants were removed and held at -80° C until assay. Total protein amount was quantified using an RCDC protein assay kit (Bio- Rad, Hercules, CA).

**Two-Dimensional Gel Electrophoresis (2-DGE):** Protein extracts (175 µg) were loaded onto pH 3-10 non-linear gradient 17 cm IPG strips (Bio-Rad) by passive re-hydration for a period of 14 hrs. Isoelectric focusing was performed using a Protean IEF Cell (Bio-Rad) subjected to 10,000 volts for a total of 60,000 volt-hours. Second-dimensional separation was performed using 8-16% pre-cast gradient polyacrylamide gels (BioRad). The gels were run at 350 volts until bromophenol blue dye disappeared. Gels were then fixed with 10% methanol, 7% acetic acid for 1hr, then stained overnight in SYPRO Ruby (Invitrogen, Carlsbad, CA), and washed in water for 1 hour before imaging. The reproducibility of 2-DGE was evaluated by preparing 3 replicate gels (n=3) for each condition.

**Differential Image Analysis:** Image analysis was performed using Progenesis software (Nonlinear Dynamics, Newcastle-upon-Tyne, UK). Automatic analysis was used to create average gels, and to perform spot detection and spot matching. Manual editing was used to edit spots. A two-fold increase or decrease was used as the cut-off value. Only unmatched spots with at least a two-fold decease or increase were selected for protein identification.

**Image Capture and Spot Picking:** Gels were imaged using a PerkinElmer Life Sciences Proexpress Proteomic Imaging System (PerkinElmer, Boston, MA) using optimized excitation (480/80) and emission (650/150) filters for SYPRO Ruby Protein Gel Stain. Gel plugs from protein spots were excised by direct picking using a ProXcision spot picking robot (PerkinElmer) equipped with a CCD camera and filter sets for SYPRO Ruby.

**Trypsin Digestion and MALDI-TOF Spectrometry:** Gel pieces were placed in a ZipPlate (Millipore, Billerica, MA) and processed as described in the manufacturer’s protocol. In brief, the gel plug was washed in 25 mM ammonium bicarbonate/5% acetonitrile for 30 minutes, and de-stained by two 30 min incubations in ammonium bicarbonate/50% acetonitrile. Gel plugs were then dehydrated with 100% acetonitrile for 15 minutes, re-hydrated in 15µl of 25 mM ammonium bicarbonate containing 150 ng Trypsin Gold (Promega, Madison, WI), and then incubated at 30^o^C overnight. The C_18_ resin of the ZipPlate was then activated with 9µl acetonitrile for 15 minutes at 37° C. Peptides were washed out of the gel plug with 180µl 0.1% trifluoroacetic acid (TFA) for 30 minutes and then bound to the C_18_ resin by low vacuum, followed by washing twice with 100µl TFA under high vacuum. Peptides were then directly eluted onto a disposable MALDI target plate (PerkinElmer) by direct vacuum elution with 10mg/ml matrix α-cyano-4-hydroxy cinnamic acid (α-CHCA; LaserBiolabs; Sophid-Antipolis Cedex, France) in 50% acetonitrile/50% TFA. The matrix was allowed to air-dry, enabling crystals to form. The MALDI plate was then loaded into a PRO-TOF 2000 MALDI-TOF (PerkenElmer). The instrument was calibrated using a two point calibration method from a peptide calibration mix (LaserBiolabs). Sample data were acquired with a mass range of 750–4500 Da. Proteins were identified by searching a local copy of the NCBI protein database (National Center for Biotechnology Information, www.ncbi.nih.gov) using the ProFound search engine (Rockefeller University, New York, NY).

**FIGURE LEGENDS**

Figure S1: Animal grouping and experimental time schedule for *P.gingivalis* expeiments*.* Four week old male ApoE^+/-^-TLR2^+/+^, ApoE^+/-^-TLR2^+/-^ and ApoE^+/-^-TLR2^-/-^ mice were fed either a HFD or a regular chow diet for 6 weeks (n=8), then inoculated once per week for 24 weeks with 50µl of either vehicle (normal saline) or 10^7^CFU) *P. g* while maintained on the chosen diet. Thus, there were 4 groups for each genotype of mice: Group 1 was fed a standard chow diet and inoculated weekly with 50µl saline vehicle (CS); Group 2 was fed a standard chow diet and inoculated with 50µl (10^7^CFU) *P. g*. (CP); Group 3 was fed a high fat diet and inoculated with 50µl saline vehicle (HS); Group 4 was fed a high fat diet and inoculated with 50µl (10^7^CFU) *P. g* (HP). In summary, mice (n=8) in each group received 24 tail vein injections of either vehicle or *P. g* once weekly.

Figure S2: Animal grouping and experimental time schedule for FSL-1expeiments*.* Effects of FSL-1 were tested in two sets of experiments. In the first, four week old male ApoE^+/-^-TLR2^+/+^ were fed either a HFD or a regular chow diet for 6 weeks (n=10) then inoculated once per week for 24 weeks with 50µl of either vehicle (normal saline) or 5µg FSL-1 while maintained on the chosen diet. The resulting 4 groups were: Group 1a was fed a standard chow diet and inoculated with 50µl saline vehicle (CS); Group 2a was fed a standard chow diet and inoculated weekly with 50µl (5µg) FSL-1; Group 3a was fed a high fat diet and inoculated weekly with 50µl saline vehicle (HS); Group 4a was fed a high fat diet and inoculated with 50µl (5µg) FSL-1. All groups were tested after 24 weeks of their diet and inoculation regimens. For the second set of experiments, four week old ApoE^+/-^-TLR2^-/-^ mice maintained on only a regular chow diet for 6 weeks (n=10) , then were divided into 2 groups: Group 1b was inoculated weekly with 50µl vehicle saline (CS); Group 2b was inoculated weekly with 50µl (5µg) FSL-1. All groups were tested after 24 weeks of inoculations.

Figure S3: TLR2 activation through FSL-1 demonstrated similar expression of increased proinflamatory cytokines as compared chow fed and injected with *P. g* in ApoE^+^/^-^-TLR2^+/+^ mice*.* Serum cytokine levels (pg/ml) in ApoE^+/-^-TLR2^+/+^ mice fed a standard chow diet and injected weekly with *P. g* or FSL-1. Data represent mean + SD.

Figure S4: : TLR2 activation through FSL-1 demonstrated similar expression of increased proinflamatory cytokines as compared high fat fed and injected with *P. g* in ApoE^+^/^-^-TLR2^+/+^ mice. Serum cytokine levels (pg/ml) in ApoE^+/-^-TLR2^+/+^ mice fed a high fat diet and injected weekly with *P. g* or FSL-1. Data represent mean + SD.

**REFERENCES:**

1. Bourassa PA, Milos PM, Gaynor BJ, Breslow JL, Aiello RJ (1996) Estrogen reduces atherosclerotic lesion development in apolipoprotein E-deficient mice. Proc Natl Acad Sci U S A 93: 10022-10027.

2. Gibson FC, III, Hong C, Chou H-H, Yumoto H, Chen J, et al. (2004) Innate Immune Recognition of Invasive Bacteria Accelerates Atherosclerosis in Apolipoprotein E-Deficient Mice. Circulation 109: 2801-2806.

3. Wank HA LM, Rose LF,Cohen DW ( 1976 Dec;) A quantitative measurement of bacteremia and its relationship to plaque control. J Periodontol 47: :683-686. .

4. Gangloff SC, Guenounou M (2004) Toll-like receptors and immune response in allergic disease. Clin Rev Allergy Immunol 26: 115-125.

5. Bhanji S WB, Sheller B, Elwood T, Mancl L. ( 2002 Jul-Aug;) Transient bacteremia induced by toothbrushing a comparison of the Sonicare toothbrush with a conventional toothbrush. Pediatr Dent 24:: 295-299.

6. Hajishengallis G, Sharma A, Russell MW, Genco RJ (2002) Interactions of oral pathogens with toll-like receptors: possible role in atherosclerosis. Ann Periodontol 7: 72-78.

7. Hajishengallis G SA, Russell MW, Genco RJ (2002 Dec;) Interactions of oral pathogens with toll-like receptors: possible role in atherosclerosis.

. Ann Periodontol 7 72-78.

8. Albrecht I, Tapmeier T, Zimmermann S, Frey M, Heeg K, et al. (2004) Toll-like receptors differentially induce nucleosome remodelling at the IL-12p40 promoter. EMBO Rep 5: 172-177.

9. Palinski W, Ord VA, Plump AS, Breslow JL, Steinberg D, et al. (1994) ApoE-deficient mice are a model of lipoprotein oxidation in atherogenesis. Demonstration of oxidation-specific epitopes in lesions and high titers of autoantibodies to malondialdehyde-lysine in serum. Arterioscler Thromb 14: 605-616.

10. Madan M, Bishayi B, Hoge M, Messas E, Amar S (2007) Doxycycline affects diet- and bacteria-associated atherosclerosis in an ApoE heterozygote murine model: Cytokine profiling implications. Atherosclerosis 190: 62-72.

11. Plump AS, Smith JD, Hayek T, Aalto-Setala K, Walsh A, et al. (1992) Severe hypercholesterolemia and atherosclerosis in apolipoprotein E-deficient mice created by homologous recombination in ES cells. Cell 71: 343-353.

12. Mallat Z, Corbaz A, Scoazec A, Graber P, Alouani S, et al. (2001) Interleukin-18/Interleukin-18 Binding Protein Signaling Modulates Atherosclerotic Lesion Development and Stability. Circ Res 89: 41e-45.
