# Supplementary figures and images for: Toll-Like Receptor-2 Mediates Diet and/or Pathogen Associated Atherosclerosis: Proteomic Findings
Source: PLoS One. 2008 Sep 12;3(9):e3204. doi: 10.1371/journal.pone.0003204 (PMC2527517; doi:10.1371/journal.pone.0003204)

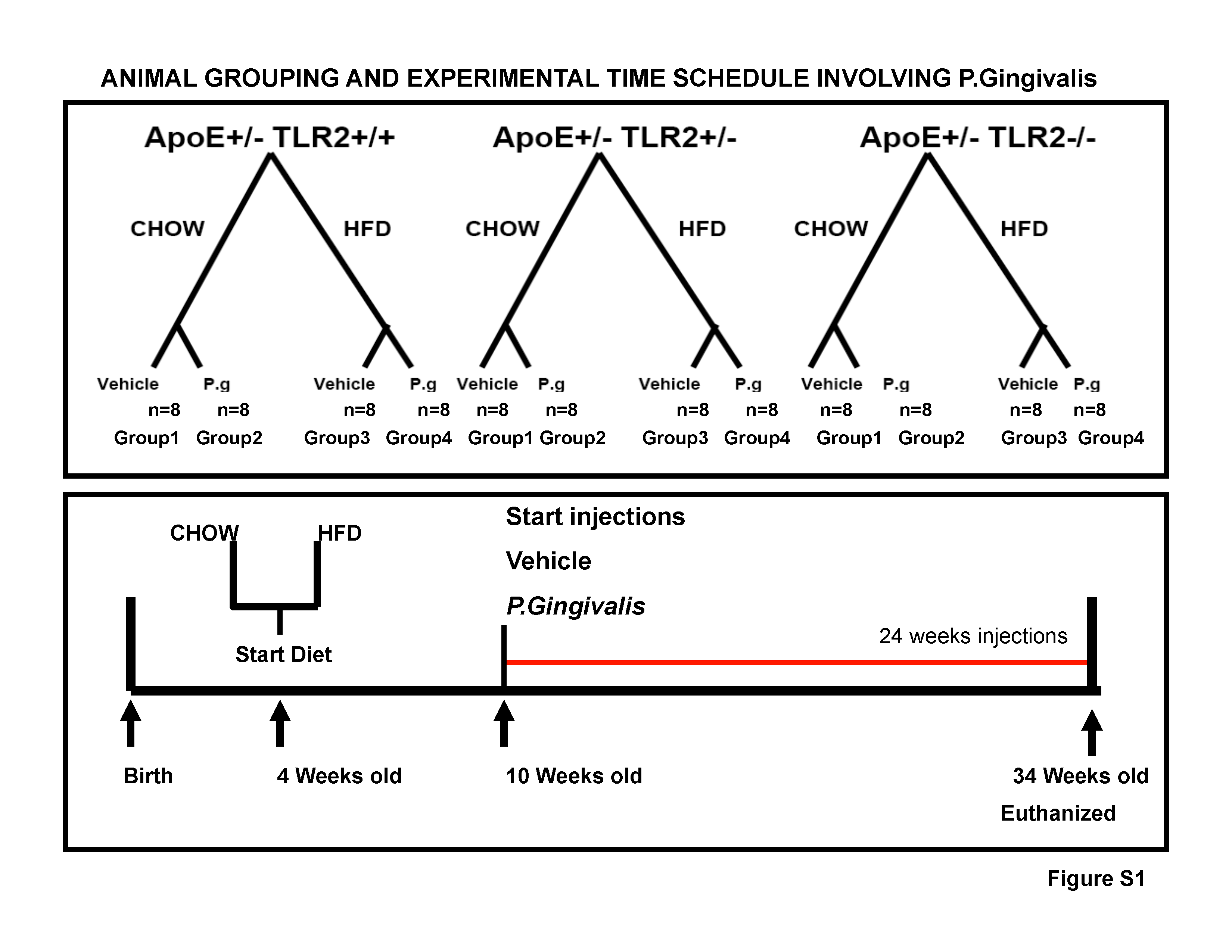

Supplement: Figure S1 — Animal grouping and experimental time schedule for P.gingivalis expeiments. Four week old male ApoE+/−-TLR2+/+, ApoE+/−-TLR2+/− and ApoE+/−-TLR2−/− mice were fed either a HFD or a regular chow diet for 6 weeks (n = 8), then inoculated once per week for 24 weeks with 50 µl of either vehicle (normal saline) or 107 CFU) P. g while maintained on the chosen diet. Thus, there were 4 groups for each genotype of mice: Group 1 was fed a standard chow diet and inoculated weekly with 50 µl saline vehicle (CS); Group 2 was fed a standard chow diet and inoculated with 50 µl (107 CFU) P. g. (CP); Group 3 was fed a high fat diet and inoculated with 50 µl saline vehicle (HS); Group 4 was fed a high fat diet and inoculated with 50 µl (107 CFU) P. g (HP). In summary, mice (n = 8) in each group received 24 tail vein injections of either vehicle or P. g once weekly. (0.83 MB TIF) [file pone.0003204.s002.tif]

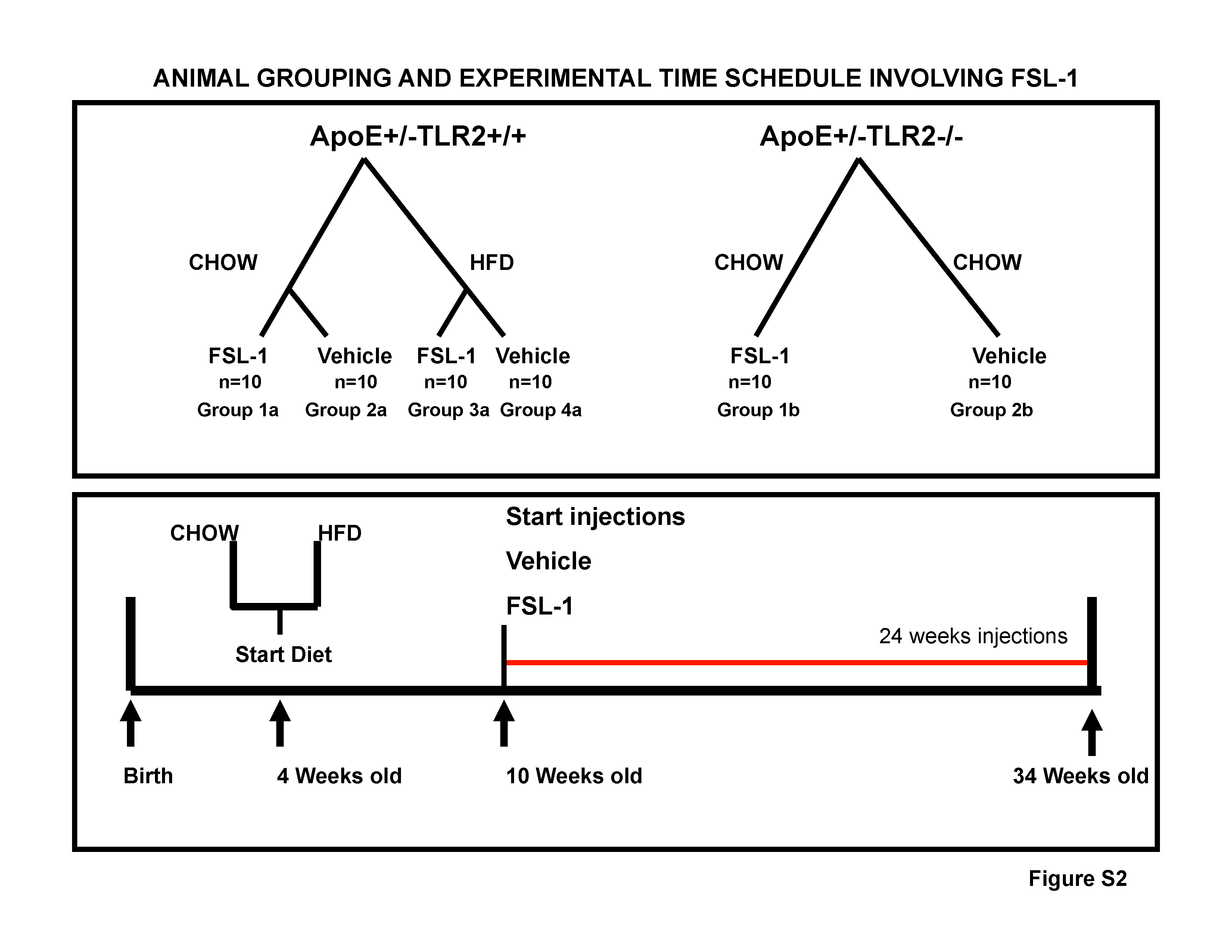

Supplement: Figure S2 — Animal grouping and experimental time schedule for FSL-1expeiments. Effects of FSL-1 were tested in two sets of experiments. In the first, four week old male ApoE+/−-TLR2+/+ were fed either a HFD or a regular chow diet for 6 weeks (n = 10) then inoculated once per week for 24 weeks with 50 µl of either vehicle (normal saline) or 5 µg FSL-1 while maintained on the chosen diet. The resulting 4 groups were: Group 1a was fed a standard chow diet and inoculated with 50 µl saline vehicle (CS); Group 2a was fed a standard chow diet and inoculated weekly with 50 µl (5 µg) FSL-1; Group 3a was fed a high fat diet and inoculated weekly with 50 µl saline vehicle (HS); Group 4a was fed a high fat diet and inoculated with 50 µl (5 µg) FSL-1. All groups were tested after 24 weeks of their diet and inoculation regimens. For the second set of experiments, four week old ApoE+/−-TLR2−/− mice maintained on only a regular chow diet for 6 weeks (n = 10), then were divided into 2 groups: Group 1b was inoculated weekly with 50 µl vehicle saline (CS); Group 2b was inoculated weekly with 50 µl (5 µg) FSL-1. All groups were tested after 24 weeks of inoculations. (0.53 MB TIF) [file pone.0003204.s003.tif]

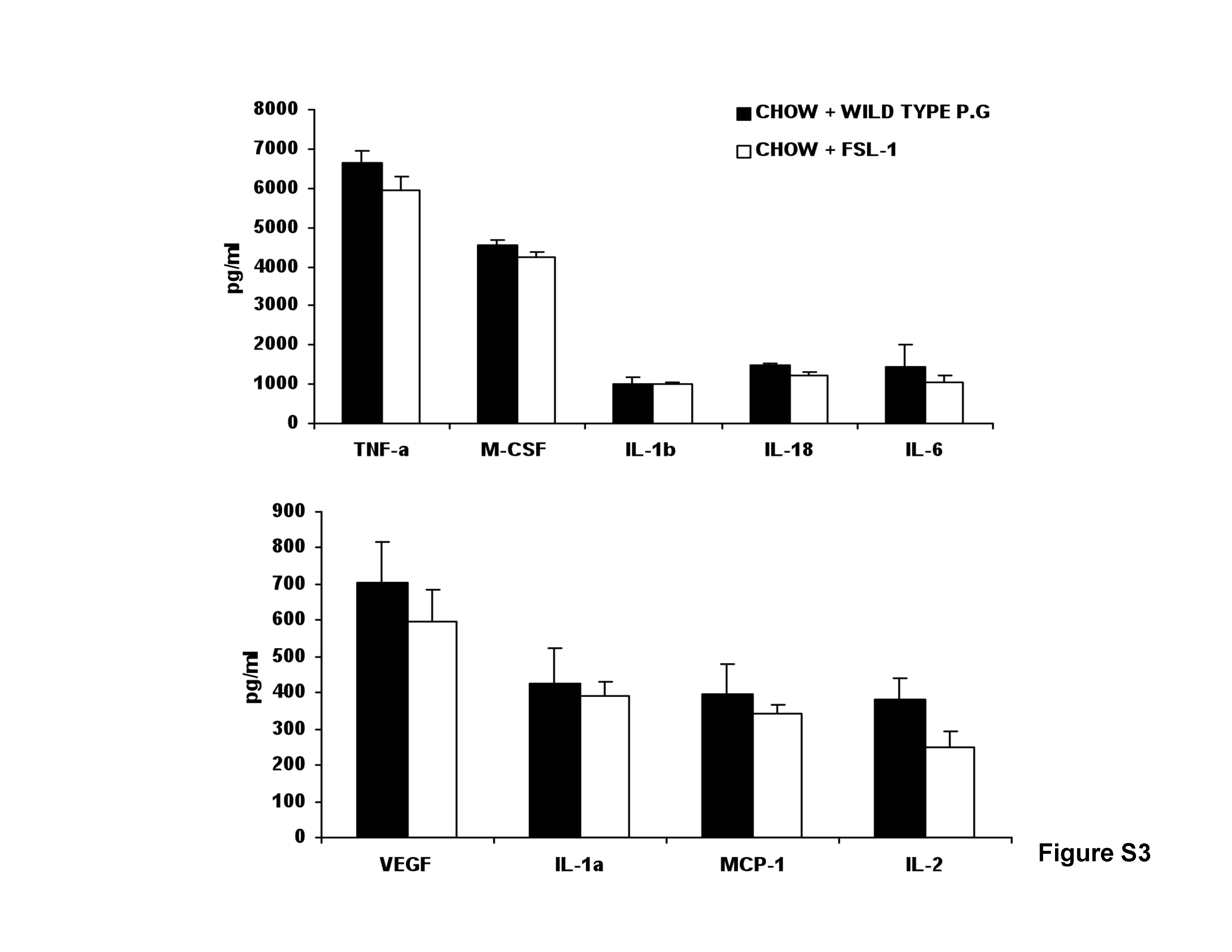

Supplement: Figure S3 — TLR2 activation through FSL-1 demonstrated similar expression of increased proinflamatory cytokines as compared chow fed and injected with P. g in ApoE+/−-TLR2+/+ mice. Serum cytokine levels (pg/ml) in ApoE+/−-TLR2+/+ mice fed a standard chow diet and injected weekly with P. g or FSL-1. Data represent mean+SD. (0.78 MB TIF) [file pone.0003204.s004.tif]

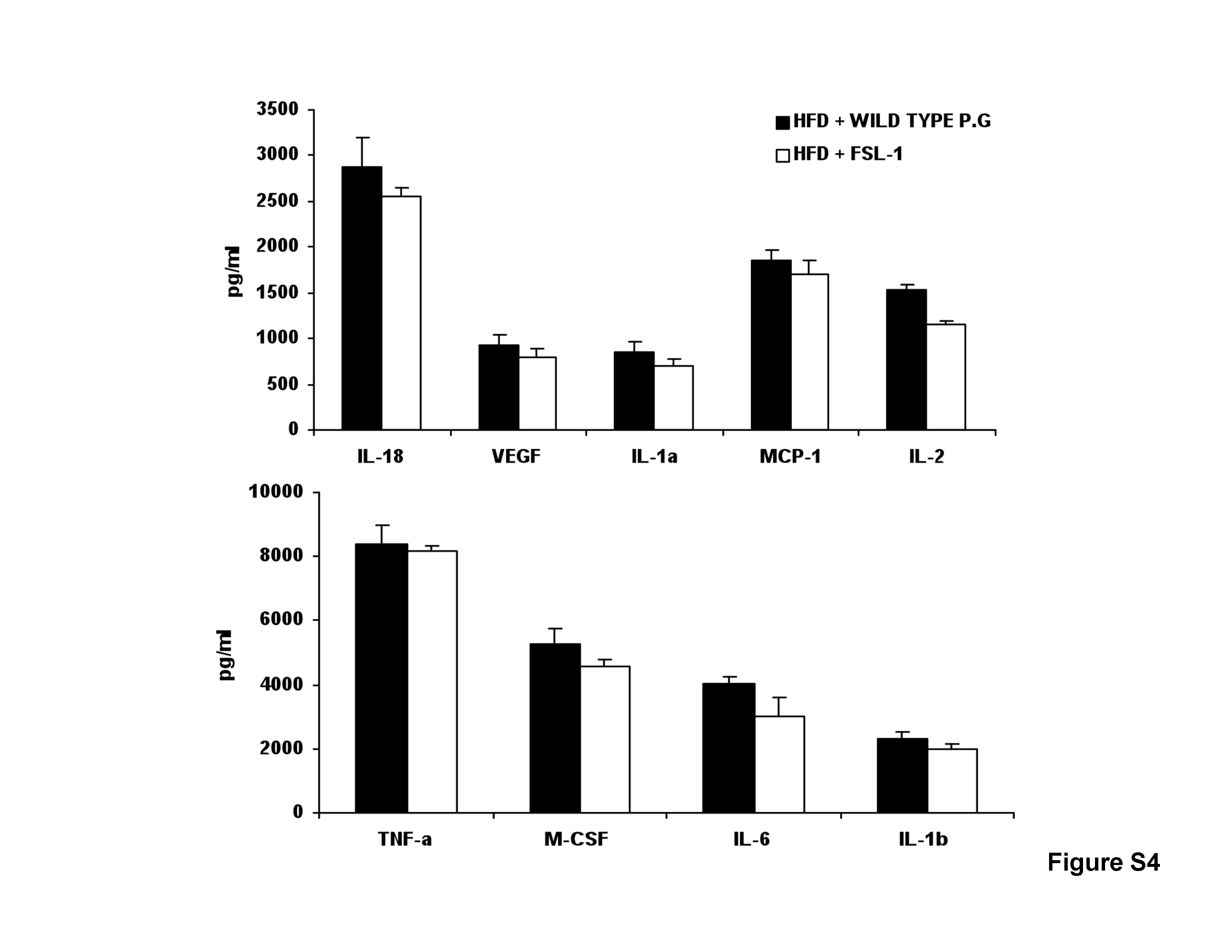

Supplement: Figure S4 — TLR2 activation through FSL-1 demonstrated similar expression of increased proinflamatory cytokines as compared high fat fed and injected with P. g in ApoE+/−-TLR2+/+ mice. Serum cytokine levels (pg/ml) in ApoE+/−-TLR2+/+ mice fed a high fat diet and injected weekly with P. g or FSL-1. Data represent mean+SD. (0.77 MB TIF) [file pone.0003204.s005.tif]
