# Supplementary material for: Toll-Like Receptor-2 Mediates Diet and/or Pathogen Associated Atherosclerosis: Proteomic Findings
Source: PLoS One. 2008 Sep 12;3(9):e3204. doi: 10.1371/journal.pone.0003204 (PMC2527517; doi:10.1371/journal.pone.0003204)
Supplement: Table S1 — Metabolic profiles of ApoE+/−-TLR2+/+, ApoE+/−-TLR2+/− and ApoE+/−-TLR2−/− mice maintained on a standard lab chow diet or a high fat diet, and injected weekly with either saline or with P. g, at 24 weeks. *Significance between ApoE+/−-TLR2+/+, ApoE+/−-TLR2+/− and ApoE+/−-TLR2−/− for respective groups. Abbreviations are as defined in the text. (0.07 MB DOC) [file pone.0003204.s006.doc]

**Table-S1 METABOLIC PROFILE OF ANIMALS**

1. Serum lipids and glucose level in mice fed with chow diet and receiving vehicle (normal saline)

|  | **TLR2^+/+^ TLR2^+/-^** | **TLR2^-/-^** | **TLR2^+/+^ TLR2^+/-^** | **TLR2^-/-^** |
| --- | --- | --- | --- | --- |
| Duration (weeks) | 14 14 | 14 P value* | 24 24 | 24 P value* |
| Number of mice (n) | 8 8 | 8 | 8 8 | 8 |
| Weight (g) | 29.1±4 28.4±7 | 29.8±7 | 32.3±5 34.2±8 | 31.9±6 |
| Total cholesterol (mg/dl) | 133.2±32 102±4 | 101.2±4 >0.05 | 138.8±24 115±24 | 111±12 >0.05 |
| HDL (mg/dl) | 76.2±5 65±4 | 85.2±7 >0.05 | 72.3±6 63.1±6 | 66.1±8 >0.05 |
| LDL (mg/dl) | 30.4±3 29±3 | 26.4±4 >0.05 | 31.5±4 34.2±3 | 30.1±3 >0.05 |
| Glucose (mg/dl) | 72.5±11 74 ± 6 | 75.9±10 >0.05 | 73.4±16 75.1±8 | 76.3±3 >0.05 |

2. Serum lipids and glucose level in mice fed with chow diet and receiving P. g.

|  | **TLR2^+/+^ TLR2^+/-^** | **TLR2^-/-^** | **TLR2^+/+^ TLR2^+/-^** | **TLR2^-/-^** |
| --- | --- | --- | --- | --- |
| Duration (weeks) | 14 14 | 14 P value* | 24 24 | 24 P value* |
| Number of mice (n) | 8 8 | 8 | 8 8 | 8 |
| Weight (g) | 31.1±9 30.8 ± 11 | 33.1 ± 4 | 30.7±5 32±9 | 32.0±3 |
| Total cholesterol (mg/dl) | 205.1±30 185 ± 8 | 189.9±7 >0.05 | 178.6±14 181±10 | 187.1±4 >0.05 |
| HDL (mg/dl) | 65.4±3 68.3±3 | 72.9±5 >0.05 | 43.7±4 41.4±10 | 44.6±8 >0.05 |
| LDL (mg/dl) | 39.6±2 35.3±4 | 31.6±3 >0.05 | 68.9±23 66.5±3 | 35.5±4 >0.05 |
| Glucose (mg/dl) | 73.3±5 74.1±5 | 73.9±12 >0.05 | 79.3±16 78.1±11 | 80.3±10 >0.05 |

3. Serum lipids and glucose level in mice fed with high fat diet and receiving vehicle (normal saline)

|  | **TLR2^+/+^ TLR2^+/-^** | **TLR2^-/-^** | **TLR2^+/+^ TLR2^+/-^** | **TLR2^-/-^** |
| --- | --- | --- | --- | --- |
| Duration (weeks) | 14 14 | 14 P value* | 24 24 | 24 P value* |
| Number of mice (n) | 8 8 | 8 | 8 8 | 8 |
| Weight (g) | 32.8 ± 9 33.9±2 | 31.1± 4 | 33.1±6 34.1±4 | 32.1±4 |
| Total cholesterol (mg/dl) | 661±44 625±33 | 649.3±38 >0.05 | 984.13±34 964±32 | 933.2±26 >0.05 |
| HDL (mg/dl) | 31.1±6 38±2 | 42.2±7 >0.05 | 27.6±5 32±6 | 39.3±8 >0.05 |
| LDL (mg/dl) | 99.5±9 83±7 | 87±7 >0 .05 | 161.9±17 168±9 | 141.5±4 >0.05 |
| Glucose (mg/dl) | 125±7 111.2±6 | 110.6±9 >0.05 | 134.1±10 121.2±4 | 109.2±15 >0.05 |

4. Serum lipids and glucose level in mice fed with high fat diet and receiving P. g.

|  | **TLR2^+/+^ TLR2^+/-^** | **TLR2^-/-^** | **TLR2^+/+^ TLR2^+/-^** | **TLR2^-/-^** |
| --- | --- | --- | --- | --- |
| Duration (weeks) | 14 14 | 14 P value* | 24 24 | 24 P value* |
| Number of mice (n) | 8 8 | 8 | 8 8 | 8 |
| Weight (g) | 34.1±3 34.1± 2 | 33.1±2 | 35.1± 2 35±6 | 33.1±1.2 |
| Total cholesterol (mg/dl) | 843.67±39 783±84 | 753±59 >0.05 | 1090.13±41 1025±34 | 975±80 >0.05 |
| HDL (mg/dl) | 19.4±2 26.6±6 | 28.1±7 >0.05 | 23.2±4 27±5 | 33.2±7 >0.05 |
| LDL (mg/dl) | 113.8±6 102.2±10 | 106.2±8 >0.05 | 182.6±16 157±29 | 160.6±10 >0.05 |
| Glucose (mg/dl) | 130.3±11 121.3±5 | 112.9±14 >0.05 | 139.2±11 128±10 | 115±9 >0.05 |

Metabolic profiles of ApoE^+/-^-TLR2^+/+^, ApoE^+/-^-TLR2^+/-^ and ApoE^+/-^-TLR2^-/-^ mice maintained on a standard lab chow diet or a high fat diet, and injected weekly with either saline or with *P. g*, at 24 weeks.*Significance between ApoE^+/-^-TLR2^+/+^, ApoE^+/-^-TLR2^+/-^ and ApoE^+/-^-TLR2^-/-^ for respective groups. Abbreviations are as defined in the text.
