# Supplementary material for: Toll-Like Receptor-2 Mediates Diet and/or Pathogen Associated Atherosclerosis: Proteomic Findings
Source: PLoS One. 2008 Sep 12;3(9):e3204. doi: 10.1371/journal.pone.0003204 (PMC2527517; doi:10.1371/journal.pone.0003204)
Supplement: Table S2 — Metabolic profile of ApoE+/−-TLR2+/+ and ApoE+/−-TLR2−/− mice fed with either a standard lab chow diet or a high fat diet, and injected weekly with either P. g or FSL-1; measurements were obtained after 24 weeks of treatments. (0.04 MB DOC) [file pone.0003204.s007.doc]

**Table- S2 METABOLIC PROFILE**

|  | *ApoE+/- TLR2+/+* | | | *ApoE+/- TLR2-/-* | | | *ApoE+/- TLR2+/+* | | |
| --- | --- | --- | --- | --- | --- | --- | --- | --- | --- |
| Diet | CHOW | |  | CHOW | |  | HFD | |  |
| Treatment | *P. g* | FSL-1 |  | *P. g* | FSL-1 |  | *P. g* | FSL-1 |  |
| Duration (weeks) | 24 | 24 | P value* | 24 | 24 | P value* | 24 | 24 | P value * |
| Number of mice (n) | 10 | 10 | 10 | 10 | 10 | 10 | 10 | 10 | >0.05 |
| Weight (g) | 30.7±5 | 32±9 | >0.05 | 32.0±3 | 30.0±5 | >0.05 | 34.1±11 | 36.1±7 | >0.05 |
| Total cholesterol (mg/dl) | 178.6±14 | 181±10 | >0.05 | 187.1±4 | 182.1±3 | >0.05 | 832.78±41 | 822.83±31 | >0.05 |
| HDL (mg/dl) | 43.7±4 | 41.4±10 | >0.05 | 44.6±8 | 41.4±7 | >0.05 | 21.4±5 | 23.4±4 | >0.05 |
| LDL (mg/dl) | 68.9±23 | 66.5±3 | >0.05 | 35.5±4 | 33.5±6 | >0.05 | 103.8±7 | 110.8±3 | >0.05 |
| Glucose (mg/dl) | 79.3±16 | 78.1±11 | >0.05 | 80.3±10 | 82.3±9 | >0.05 | 123.7±9 | 120.6±5 | >0.05 |

Metabolic profile of ApoE^+/-^-TLR2^+/+^ and ApoE^+/-^-TLR2^-/-^ mice fed with either a standard lab chow diet or a high fat diet, and injected weekly with either *P. g* or FSL-1; measurements were obtained after 24 weeks of treatments.
